# Influence of the load exerted over a forearm crutch in spatiotemporal step parameters during assisted gait: pilot study

**DOI:** 10.1186/s12938-018-0527-z

**Published:** 2018-07-18

**Authors:** Carmen Ridao-Fernández, Gema Chamorro-Moriana, Joaquín Ojeda

**Affiliations:** 1 Department of Physiotherapy, Research Group “Area of Physiotherapy CTS-305”, University of Seville, C/Avicena s/n, C.P. 41009 Seville, Spain; 2 Department of Mechanical Engineering and Manufacture, Research Group “Mechanical Engineering”, University of Seville, Seville, Spain

**Keywords:** Assisted walking, Forearm crutches, Load, Step parameters

## Abstract

**Background:**

Assisted gait with forearm crutches is frequently performed during the recovery of musculoskeletal injuries of the lower limb. The amount of body weight applied to the crutch or crutches depends on the pathology and the treatment phase. The transition from assisted gait with two crutches to a single crutch is usually recommended when the subject is able to load the 50% of the body weight upon the affected member. An altered assisted gait will cause biomechanic alterations and, therefore, longer treatments and relapses. The aim of this study was to analyze the influence of 10, 25 and 50% of body weight applied to a forearm crutch during a unilateral assisted gait in the spatial and temporal step parameters to determine the load that produces alterations in gait biomechanics and the load that does not.

**Methods:**

Eleven healthy subjects performed normal gait (NG) and assisted gait with a forearm crutch, in which the applied loads were: comfortable (C), 25 and 50% of their body weight. Vicon System was employed for gait recording. GCH System 2.0 and GCH Control Software 1.0 controlled the loads. The variables were: step length, step period, velocity, step width and step angle. Friedman test compared all the gait modalities: NG and the different loads. Wilcoxon signed-rank test analyzed ipsilateral and contralateral step parameters to the crutch globally and for each subject.

**Results:**

Friedman test showed significant differences between NG, C, 25 and 50%, especially for step period and velocity. Wilcoxon test had significant differences only in 4 of the 20 general comparisons between ipsilateral and contralateral steps to the crutch. In the analysis by subjects, step length, step period and velocity showed 79/132, 110/132 and 58/66 significant differences, respectively.

**Conclusions:**

The increase in the load exerted over a forearm crutch produced an increase in the step period, accompanied by a reduction of step length and gait velocity. Step width and step angle were not modified. The unloading of 25 and 50% of body weight on a single crutch is incorrect from the biomechanical point of view. Two crutches should be employed when the body weight to unload exceeds 10%.

**Electronic supplementary material:**

The online version of this article (10.1186/s12938-018-0527-z) contains supplementary material, which is available to authorized users.

## Background

Gait is one of the most important functions of human locomotion [1], and its reeducation is an essential part of many physiotherapy treatments [2, 3]. That is why this motor function has been analyzed from many perspectives: biomechanics, innovative technology and clinical procedures [4–6]. The three aspects have been integrated in this research.

Assisted gait with forearm crutches is frequently used in the clinical setting. Moreover, a large number of musculoskeletal lower member injuries require partial unloads during their recovery [3, 7–9]. The amount of body weight unloaded over the crutch or crutches will depend on the pathology and the recovery phase in which the subject is [10, 11]. Regarding the transition from assisted gait with two crutches to a single crutch, different recommendations exist. Some authors recommend this change takes place when the subject is able to apply 50% of the body weight upon the affected member [7]. An altered assisted gait produces corporal misalignments which generate muscular alterations and articular overloads, amongst others [12, 13]. Therefore, longer treatments as well as relapses may occur.

Gait biomechanics is frequently modified by the presence of pathology [2]. Therefore, to know the isolated influence of the load on gait, it is necessary to avoid the interference of the pathology [14]. Moreover, only a sample composed of healthy subjects allows the use of different levels of load, and the assisted gait without crutches.

The analysis of the parameters that influence gait will allow us to prevent and correct the alterations of this function [15, 16]. The studied parameters, related to step, are: length, period, width and angle; in addition to gait velocity. These parameters are essential and serve to evaluate the functional ability of the subject [17, 18]. Besides, they are part of a great number of functional gait assessment scales relevant in the clinical setting, such as *Gait and Balance Scale* [4], *Tinetti Mobility Test* [19] *and Chamorro Assisted Gait Scale* [3].

Moreover, the spatiotemporal step parameters have been previously studied in assisted gait with forearm crutches [20]. However, they have never been analyzed when comparing different levels of load. That is why it is very difficult to establish protocolized treatments to reeducate gait, initially assisted by crutches and reducing the load until achieving normal gait [3]. The need to make a correct and optimal assisted gait [21], and to increase the scientific evidence of gait reeducation in the clinical setting [22] leads to the objective of this study: to analyze the influence of 10, 25 and 50% of body weight applied to a forearm crutch in the spatial and temporal step parameters during an unilateral assisted gait to determine the load that produces alterations in gait biomechanics and the load that does not. Regarding the study objective, the hypothesis of the authors was: the increase of the load applied to a forearm crutch leads to a modification of the step parameters, especially step length, step period and gait velocity.

## Methods

### Participants

The sample was composed of 11 Caucasian healthy subjects from south-west Europe (4 men and 7 women), with an intermediate socio-cultural and socio-economic status. Subjects were aged between 21 and 53 years (mean ± SD: 32 ± 10.9 years).

The inclusion criteria were: aged between 18 and 60 years old; previous experience with crutches (to have employed crutches due to a musculoskeletal injury) and normal gait (defined by Kim et al. [23] as asymptomatic with free cadence on walking). As additional inclusion criterium, the subjects were asked to pass a simple static previously employed equilibrium test [24] which consisted of maintaining monopodal balance for 30 s on each foot without suffering any great bodily movements (overacted movement, mainly arms, time and a motor skill to rebalance).

The exclusion criterion was: an evident general coordination and physical ability disorder that could alter the normal or assisted gait, such as history of vestibular or neurological disorders, proprioceptive musculoskeletal alterations or cerebellar tumors, among others.

### Data collection

The Vicon^®^ System (Oxford, UK) of three-dimensional motion analysis (3D) [25], the instrumented crutches called GCH 2.0 load measurement system (University of Seville, Seville, Spain) for assisted gait [26], and the GCH Control Software 1.0 (University of Seville, Seville, Spain) [27] were used in this research.

**Fig. 1:**
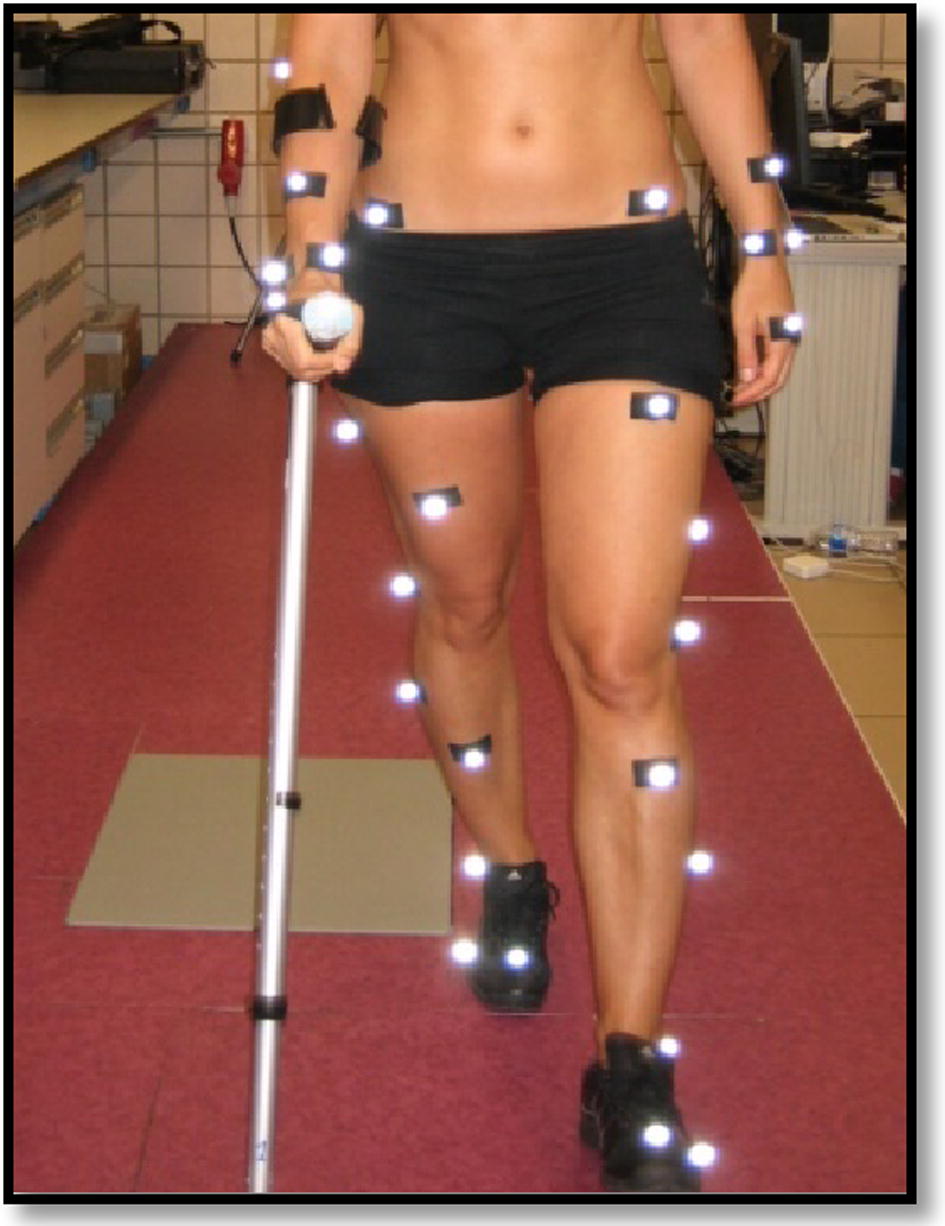
Assisted gait with simultaneous heel and crutch support at two points

Data collection was carried out under laboratory conditions (with the same artificial light and temperature) on an 8.5-m-long walkway. The participants performed assisted gait at two points, with simultaneous support for heel and crutch. A height that corresponded to an elbow flexion of 20°–30° was selected for the crutch [28]. The participants performed the walk at free cadence, and completed the corridor on 10 occasions for each gait modality. First, they performed gait without crutches, called normal gait (NG); and then unilateral assisted gait (UAG) with an elbow crutch. NG modality was included in the study in the absence of pathology and without the distracting effect of the crutch. UAG was contralateral, as the unloaded member was the opposite of the crutch (see Fig. 1). The applied loads in UAG were: Comfortable (C), thus the subjects could walk in a comfortable way by applying a level of load freely chosen. It was observed that this load was close to the 10% of the subject body weight; 25 ± 5% (25%); and 50 ± 5% of the body weight or the maximum load the subject was able of applying (50%). For 25 and 50%, a margin of tolerance of 5% was established as the percentage of error allowed. Thus, all errors equal or minor than 5% were admitted. In both, 25 and 50% measurements, the assessors gave instructions to the subjects to maintain the required load within the margin of tolerance. They viewed and controlled the peak loads [24, 27] exerted on the crutches using: GCH 2.0 load measurement system [27], that measured loads; and GCH Control Software 1.0 [27]. The latter showed the applied loads on a computer screen in real time.

**Fig. 2:**
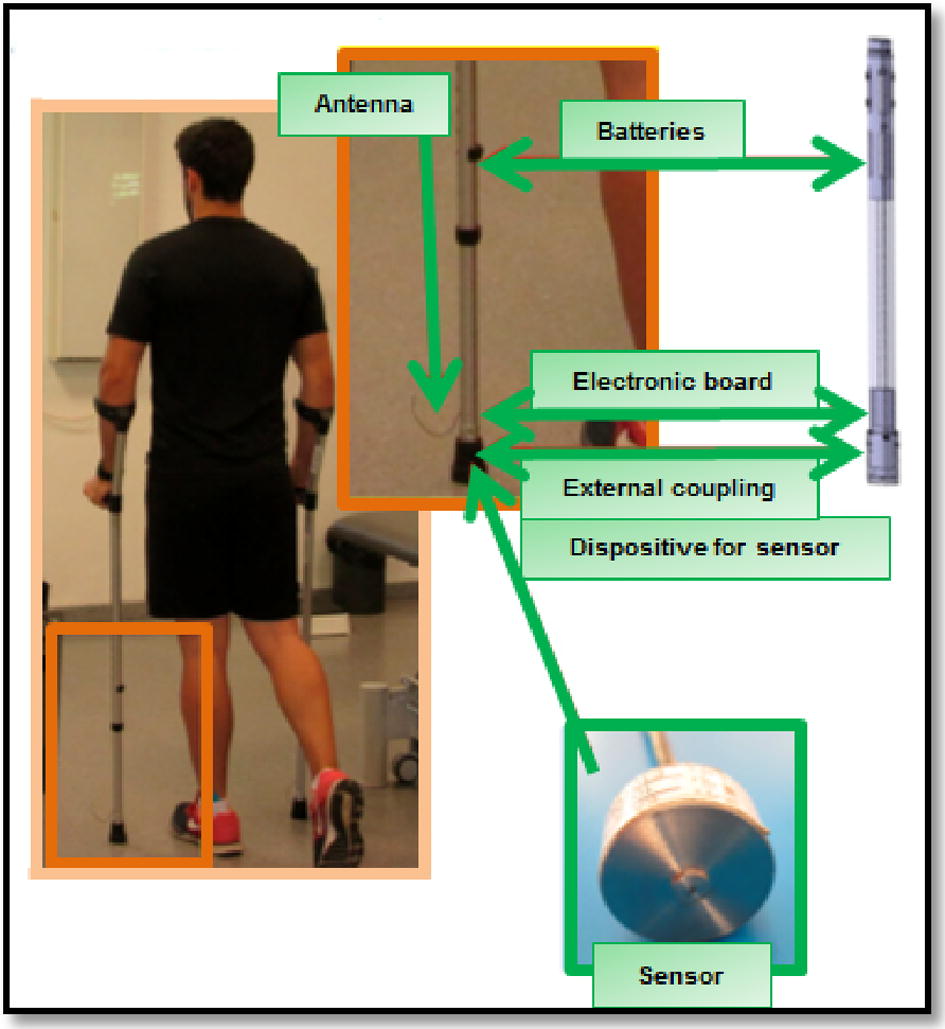
GCH 2.0 load measurement system for aided gait with forearm crutches

GCH System 2.0 measures ground reaction force acting along the crutch [24, 27]. This ground reaction force is directly proportional to the force exerted by the subject over the crutch. GCH System 2.0 contains a miniature force sensor within the distal part of a forearm crutch. This cell is connected to an electronic board and power batteries. The function of the data acquisition card is to emit signal wireless way, which is detected by a small USB receiver connected to a computer. GCH has a compact design, which integrates all these elements inside the distal part of the forearm crutch (see Fig. 2) [27].

Gait analysis was performed using a modified Newington gait model proposed by Davis et al. [25]. This protocol defines 16 markers in the lower limbs, and is based on a minimal set of markers.

A six-camera motion capture system (Vicon^®^) recorded the marker trajectories at 100 Hz. Two set of measures were carried out. First, static trials allowed the definition of local frames attached to the segments in order to estimate the position and orientation of the bodies in the space. Second, a set of 10 dynamic trials were recorded for each gait modality (NG and UAG: C, 25 and 50%). The aim of these trials was to capture one gait cycle per trial. This cycle was situated in the centre of the walkway, considered the capture volume. All the recordings were carried out in the Gait Analysis Laboratory of the Mechanical Engineering Department at the University of Seville.

In the next step, the data recorded in the trials were the input data in an inverse problem to obtain the kinematic data of the model. Davis et al. [25] defined and detailed the procedure.

**Fig. 3:**
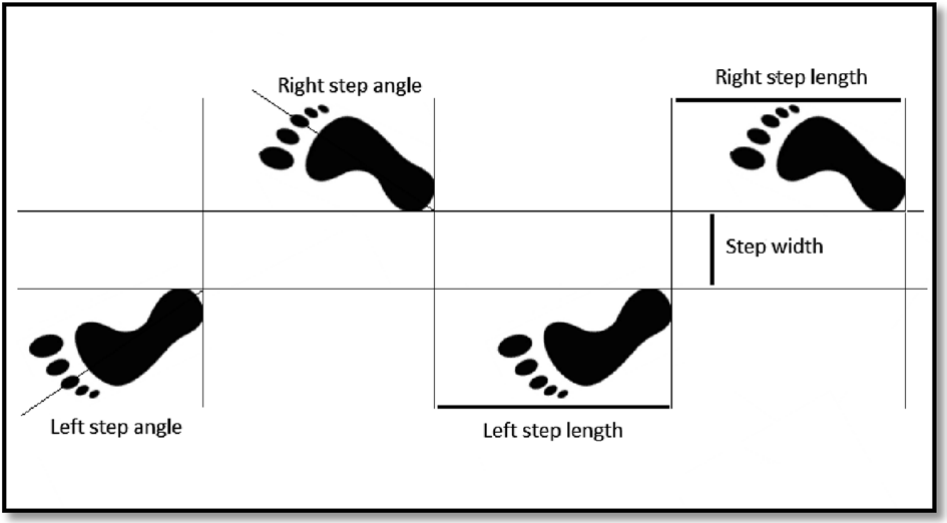
Representation of the parameters: step length, step angle and step width

The study variables were: step length, distance between one heel strike to the next one on the other side; step period, time to carry out one step; velocity, relation between the stride length and the stride period; step angle, determined by the anterior–posterior axis of the foot local frame and the line of progression; and step width, mediolateral distance between the feet and measured on the heels (see Fig. 3) [29, 30].

A routine applied to MATLAB R2014b software was developed by the authors of the study to obtain the variables of the study.

### Statistical analysis

The data were organized and analyzed using IBM SPSS Statistical Software (Version 22.0; SPSS Inc., Chicago, IL, USA). The descriptive analysis included mean, median, standard deviation (SD), minimum, maximum, and 25, 50 and 75 percentiles. Statistical analyses were carried out by means of parametric tests after performing a Shapiro-Wilks normality test to the data. NG as well as the different loads applied during UAG (C, 25 and 50%) were compared using the Friedman test of variance by ranks for related samples (p = 0.05). In the cases which showed significant differences, loads were then compared two by two (NG-C, NG-25%, NG-50%, C-25%, C-50%, 25–50%). The variables measured for ipsilateral and contralateral step of crutch in the different gait modalities performed (NG and UAG: C, 25 and 50%) were generally compared using the Wilcoxon signed-rank test for related samples (p = 0.05). Gait analysis regarding the walking modality (NG and UAG: C, 25 and 50%) for each study subject was performed using the Wilcoxon signed-rank test for related samples. Finally, the Pearson correlation index studied the relationship between each parameter for each gait modality and the age, weight and height of the subjects.

## Results

**Fig. 4:**
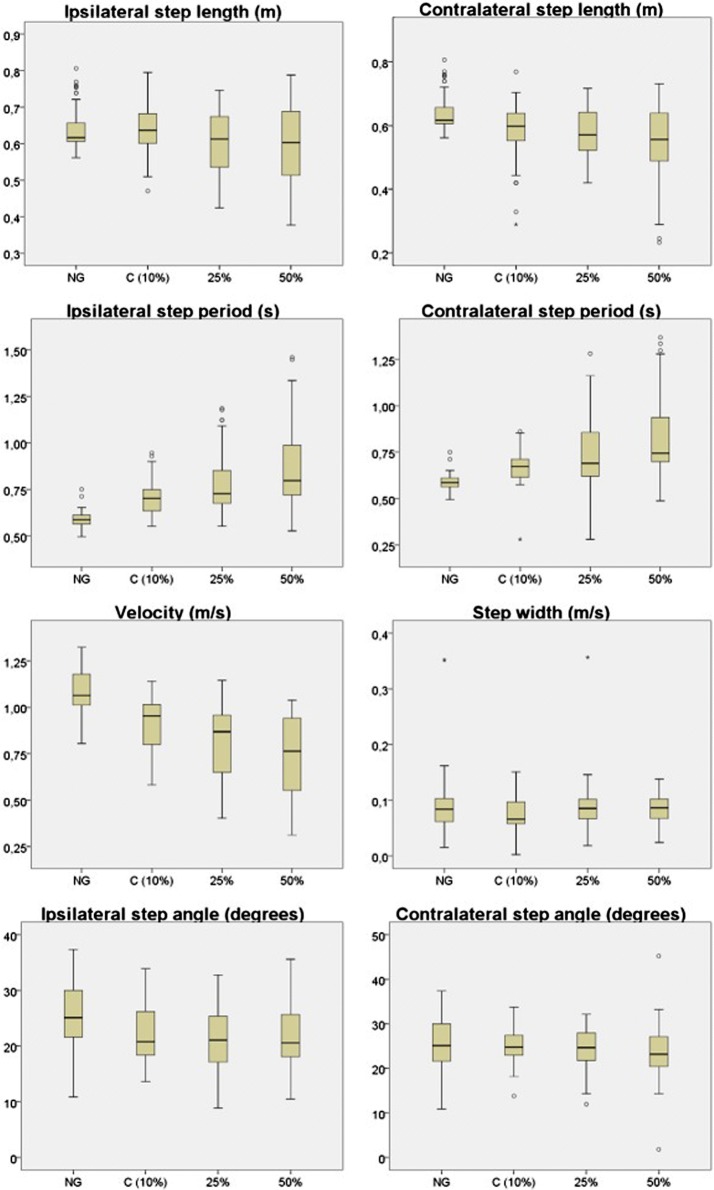
Descriptive analysis representation

The descriptive analysis of the variables *step length, step period, velocity, step angle and step width* for the NG and UAG (C, 25 and 50%), is represented in Fig. 4. The complete data is shown in Additional files 1, 2, 3, 4, 5, 6.

Figure 4 showed a reduction in step length and velocity, as well as an increase in step period when increasing the load exerted on the crutch. These changes were observed in both steps, ipsilateral and contralateral. Step width and step angle, however, did not appear to be modified in relation to the load.

Table 1 shows the results obtained in the Friedman test when comparing the loads (NG, C, 25% and 75%) two by two for all the parameters considered in this research. A Table containing the completed results is presented in Additional file 7. Additional file 8 includes a chart that represents data in Table 1.

**Table 1:**
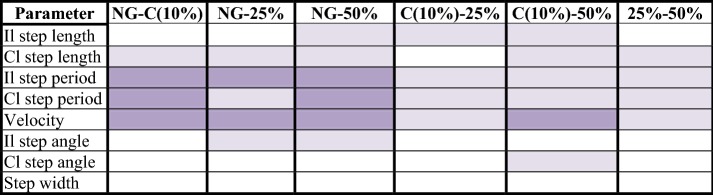
General comparisons between the loads applied to the crutch, for each study variable (difference of means)

significant confidence intervals with a high effect size (> 0.8);  significant confidence intervals with a medium effect size (> 0.3);  non-significant results

*Il* ipsilateral, *Cl* contralateral, *NG* normal gait, *C* comfortable

The general comparisons showed differences between NG and the applied loads during UAG (C, 25 and 50%). Step period and velocity always obtained significant results. Moreover, many of them were reinforced by a high effect size (> 0.8). For its part, step length obtained four non-significant results (ipsilateral step length when comparing NG-C, NG-25% and 25–50% and contralateral step length when comparing C-25%) and eight significant results. Step width was the only variable that obtained non-significant differences in every case.

In the Wilcoxon signed-rank test, significant differences were found only in 4 of the 20 comparisons made between homolateral and contralateral steps: NG step period (Ipsilateral and Contralateral), step length 25%, step length 50% and step period 50%.

Tables 2, 3, 4, 5, 6 represent the results obtained in the analysis of parameters depending on the load applied for each subject. The completed results are shown in Additional files 9, 10, 11, 12, 13 and graphically represented in Additional file 14.

**Table 2:**
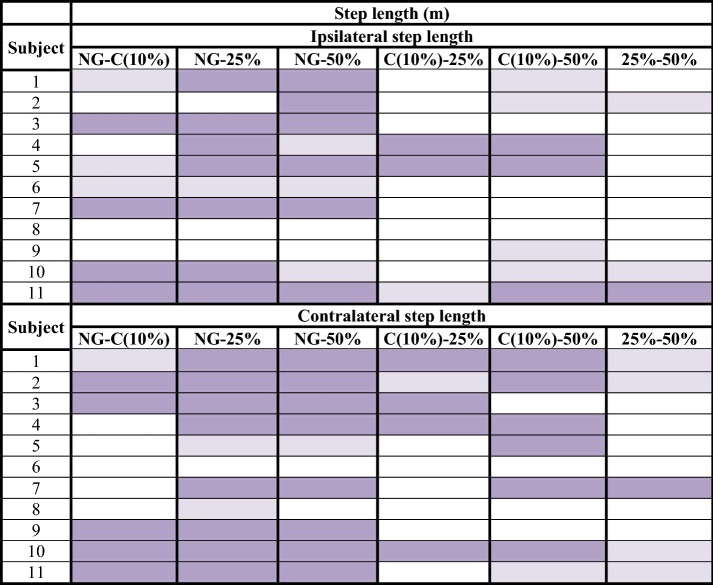
Step length analysis: difference of means between normal gait and unilateral assisted gait modalities (C, 25 and 50%)

significant confidence intervals with a high effect size (> 0.8);  significant confidence intervals with a medium effect size (> 0.3);  non-significant results

*NG* normal gait, *C* comfortable

**Table 3:**
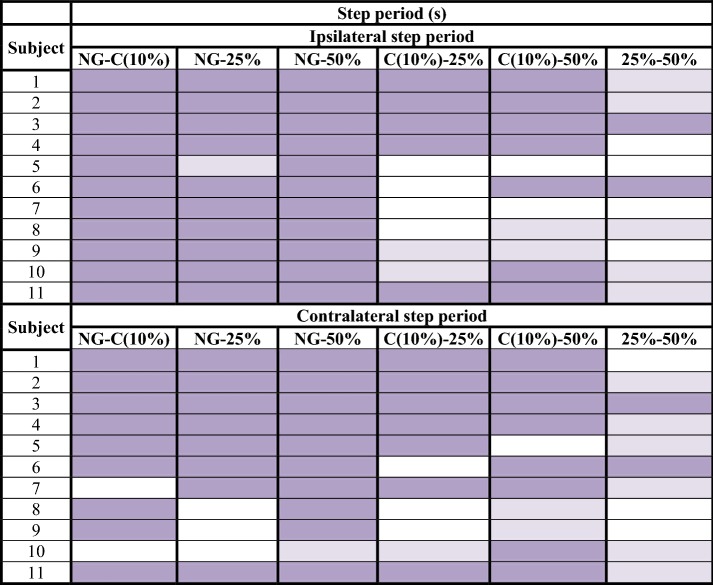
Step period analysis: difference of means between normal gait and unilateral assisted gait modalities (C, 25 and 50%)

significant confidence intervals with a high effect size (> 0.8);  significant confidence intervals with a medium effect size (> 0.3);  non-significant results

*NG* normal gait, *C* comfortable

**Table 4:**
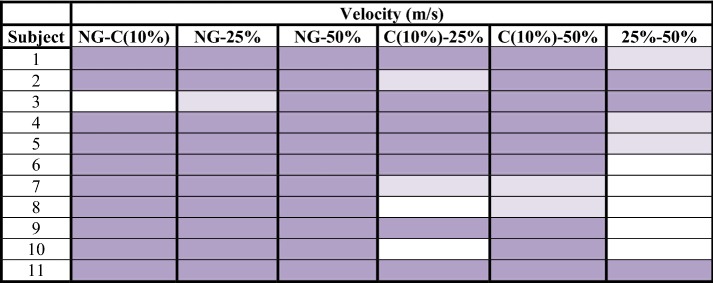
Velocity analysis: difference of means between normal gait and unilateral assisted gait modalities (C, 25 and 50%)

significant confidence intervals with a high effect size (> 0.8);  significant confidence intervals with a medium effect size (> 0.3);  non-significant results

*NG* normal gait, *C* comfortable

**Table 5:**
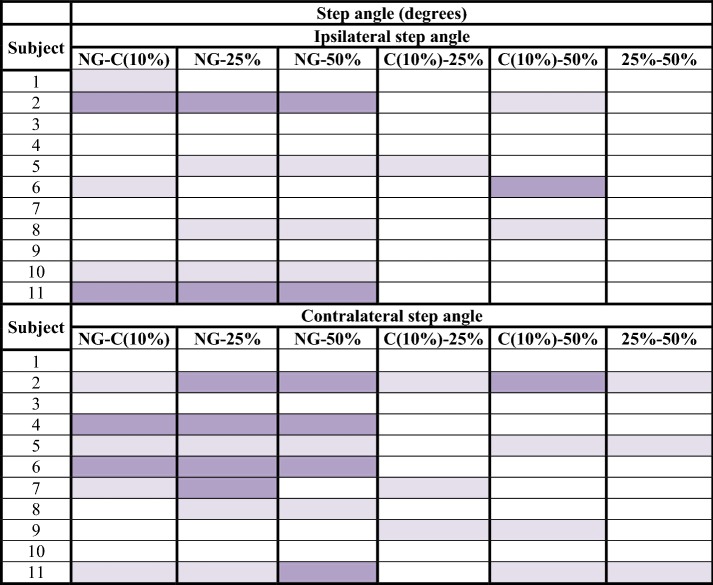
Step angle analysis: difference of means between normal gait and unilateral assisted gait modalities (C, 25 and 50%)

significant confidence intervals with a high effect size (> 0.8);  significant confidence intervals with a medium effect size (> 0.3);  non-significant results

*NG* normal gait, *C* comfortable

**Table 6:**
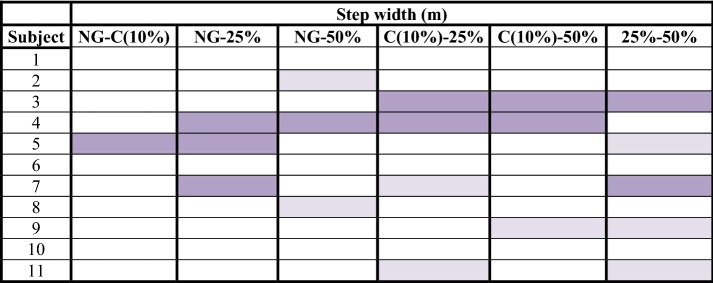
Step width analysis: difference of means between normal gait and unilateral assisted gait modalities (C, 25 and 50%)

significant confidence intervals with a high effect size (> 0.8);  significant confidence intervals with a medium effect size (> 0.3);  non-significant results

*NG* normal gait, *C* comfortable

Step length obtained 79/132 significant confidence intervals at a 99% confidence interval (Table 2). In addition, 58 of these significant values of p were accompanied by a high effect size (> 0.8). Regarding step period, 110/132 results had significant values, and 89 of them obtained a high effect size (Table 3). Gait velocity showed 58/66 significant results, 53 of them with a high effect size (Table 4). The non-significant results obtained in step length, step period and velocity focused especially on the comparison of two consecutive modalities or load levels. That is, NG-C, C-25% and 25–50%. These outcomes were: 37/53 non-significant results for step length, 16/22 for step period and all non-significant results obtained for velocity (8/8). Step angle and step width, nevertheless, showed only 48/132 and 19/66 significant results respectively.

The Pearson correlation coefficients calculated in relation to age, height and weight of subjects did not find significant results.

## Discussion

In this paper, gait in subjects that walked with a forearm crutch has been analyzed. This research focused on the step parameters as they are the reference unit in this cyclic activity. Healthy subjects were necessary to study the influence of different levels of unloading in the analyzed parameters, without the interference that a pathology could exert. The study of the unloading of different percentages of body weight over the crutch allows us to understand the corporal adaptations performed during assisted gait. These adaptations are not always beneficial, and can involve biomechanical problems, such as vertebral deviations or articular overloads, amongst others [3]. That is, an incorrect position or gesture during gait could prejudice the patient [12]. When the subjects were asked to walk comfortably, in C modality, the measurements registered by GCH 2.0 load measurement system and GCH Control Software 1.0 were very close to the 10% of their body weight.

### Adjustments to load increase. Progression from bilateral assisted gait to unilateral assisted gait

Table 1 shows the comparisons between the different loads applied on the crutch. The global analysis of the study results showed that significant differences exist between all gait modalities performed (NG, C, 25 and 50%). Step width was the only parameter that showed no differences in the global analysis when the applied load to the crutch was increased. Moreover, this parameter obtained very little differences in the study of each subject. Authors defend that this result was due to the presence of the crutch, which causes an increase on the support base. Thus, it was not necessary to have the feet apart to enhance this support base.

Step length, step period and gait velocity showed to be compensation adjustments in response to a higher request of weight unload. As is shown in the results section, step length, step period and velocity showed differences between the NG and UAG tasks. Differences in these parameters were also observed when the load applied to the crutch was increased. Thus, a reduction in step length, an increase in step period and a reduction in gait velocity were produced. Moreover, if we observe the most proximal levels of difficulty, which can be considered as consecutive load levels (NG-C, C-25% and 25–50%), we will find less differences in the three cited parameters. This fact suggests that the higher the increase on the load applied to the crutch the bigger the modification of the step parameters carried out by the subjects, thus progressively getting away from the NG pattern [3].

The current clinical tendency consists in passing from assisted gait with two crutches to assisted gait with a single crutch when the load of the 50% of body weight over the affected member is indicated. This schedule is supported by different authors [7, 31] but rejected by the authors of this study. Given the results achieved, gait reeducation was not correct or functional when the unloading of weight required was the 50% of body weight or maximum possible unloading over a single crutch. Moreover, in most cases, the subjects were not able to achieve that level of load applied to the crutch despite performing the required body compensations (center of mass deviation, crutch inclination, etc.). The evaluators requested the maximum possible discharge in the light of the inability of the subjects to download 50% of their body weight. This maximum load did not reach 40% in some participants. Other studies have described that the unloading of more than 25% of body weight over a crutch is incorrect from the biomechanical point of view [9, 32]. The 10 and 25% are two proximal levels of unloading. Nevertheless, subjects modified the spatiotemporal step parameters between both percentages. Thus, participants did not perform a functional gait pattern in the light of the requirement of the 25% of load. Besides, none of them was able to maintain a biomechanically correct gait (that is, with corporal alignment, movement fluency, simultaneous support of heel and crutch, etc.) when applying this percentage of load. When a comfortable assisted gait was required, the loads registered were very close to 10% of body weight, as shown in the “Data collection” section. For this reason, we defend that as from 10% of body weight it is necessary to add a second forearm crutch, which allows maintaining a correct and functional gait pattern, without altering the corporal alignment and the step parameters.

### Step length, step period, step angle and gait velocity

The length, the period and the angle of the step are the three parameters measured for each step, ipsilateral and contralateral to the crutch. Length was the most constant of these. Subjects modified their step length less to meet the required unloading of weight. Besides, less significant differences were observed in the ipsilateral step to the crutch. That is, the step under the member that had not been unloaded. This datum made the authors think about the asymmetry that the crutch effect produced between both steps, despite the subjects having a high level of coordination and previous experience in the use of crutches. Instead step period and velocity were the most modified parameters due to the increase of gait demand. The maximum step length and the minimum velocity were achieved when the subject unloaded 50% of their body weight. The increase of load exerted over the crutch constitutes a progression in the degree of difficulty of gait, which is compensated through the increase of step length and the decrease in velocity [33]. The changes in these parameters against the requirement of high levels of load are due to biomechanical adjustments performed as mechanisms of compensation. When an excessive force is exerted over the crutch, it is very difficult to maintain the stability of the proximal area (trunk, hip, etc.). This is due to the vertebral inclination and, therefore, the deviation of the center of gravity, which are necessary in high levels of load. The step angle, for its part, has shown a lot of dispersion. Thus, it has not followed a characteristic pattern in relation to the studied variables.

### Gait symmetry

The analysis of the ipsilateral step parameters to the crutch regarding the contralateral allowed us to study the asymmetries of UAG. This was examined in the parameters measured for both steps, that is, step length, step period and step angle. As shown in the results section, 16 of the 20 comparisons made between both steps did not show differences. Subjects had a sufficient level of coordination so as to perform the required unloading of weight without modifying one step regarding another [10]. In 2 of the 4 cases that showed differences, the load was the 50% of the body weight of the subject. They were step length 50% and step period 50%. In one of those 4 cases, the load was the 25%. It was step length 25%. Therefore, the use of the crutch modified the symmetry of step length and step period mainly when the applied load to the crutch was high and the gait was not functional. The demand of assisted gait is, therefore, greater. Despite the high level of the coordination of the subjects, a tendency to the gait asymmetry was observed during UAG. Said tendency was produced by the presence of the crutch and was considered relevant from the clinical point of view. For this reason, the physiotherapist should pay special attention to the correction of the gait symmetry, especially in less coordinated subjects and in the unloading of high percentages of body weight. Even so, as from 10% body weight, it will be necessary to add a second forearm crutch, as mentioned above. Thanks to the correction of the asymmetry, the gait reeducation process will be optimized, improving the functional recovery of the patient and avoiding relapses of the injury.

### Study limitations

Even though it is an innovative and complex research due to using different technologies, this study presents a limitation regarding the sample size. The authors propose to study the influence of the load percentage exerted over a forearm crutch on a higher number of healthy subjects to confirm the findings of this pilot study.

Besides, a study on assisted gait as a dual-task in which the crutch is carried by dominant and non-dominant hand has been recently developed [34]. The findings on both studies will be complemented in the assessment of unilateral assisted gait with a forearm crutch.

As future work, it would be interesting to study the upper limbs and the trunk parameters (pelvic dissociation, deviation of the center of mass, etc.) in the assisted gait with a unilateral forearm crutch to analyze the symmetries regarding these parameters [35].

## Conclusions

This paper showed that the increase of load exerted over a forearm crutch produces an increase in the step length, accompanied by a reduction in step period and gait velocity. Step width and step angle were not modified following the increase of weight unloaded. Further studies should be conducted to confirm the results of this pilot study. Besides, this paper has indicated that the clinical tendency to remove a forearm crutch when the load of the 50% of body weight is indicated is wrong. The unloading of 50%, and even 25% of body weight over a single crutch appears to be incompatible with a correct gait pattern. A forearm crutch should be employed when the unloading to be carried out does not exceed 10% of the body weight of the subject. In this way, a correct and optimal reeducation of assisted gait could be performed, that reduces the energy cost during its execution. Thus, it will be possible to establish protocolized treatments to reeducate a gait initially assisted by crutches by means of reducing the load until achieving normal gait. All this will encourage the recovery from the injury and will avoid the appearance of relapses. A clinical tendency to gait asymmetry was observed in UAG, due to the presence of the crutch. The symmetry between the ipsilateral and contralateral steps to the crutch should be considered by the physiotherapist to optimize gait reeducation and, therefore, the functional recovery process of the patient. The findings of this cinematic paper will be completed with the analysis of kinetic data in similar study conditions.

## Additional files

**Additional file 1.** Descriptive analysis of study variables.

**Additional file 2.** Descriptive analysis of step length.

**Additional file 3.** Descriptive analysis of step period.

**Additional file 4.** Descriptive analysis of velocity.

**Additional file 5.** Descriptive analysis of step angle.

**Additional file 6.** Descriptive analysis of step width.

**Additional file 7.** General comparisons between the loads applied to the crutch, for each study variable.

**Additional file 8.** Representation of general comparisons between the loads applied to the crutch, for each study variable.

**Additional file 9.** Step length analysis: difference of means between normal gait and unilateral assisted gait modalities (C, 25% and 50%).

**Additional file 10.** Step period analysis: difference of means between gait without crutches and unilateral assisted gait modalities (C, 25% and 50%).

**Additional file 11.** Gait velocity analysis: difference of means between gait without crutches and unilateral assisted gait modalities (C, 25% and 50%).

**Additional file 12.** Step angle analysis: difference of means between gait without crutches and unilateral assisted gait modalities (C, 25% and 50%).

**Additional file 13.** Step width analysis: difference of means between gait without crutches and unilateral assisted gait modalities (C, 25% and 50%).

**Additional file 14.** Representation of the difference between gait without crutches and unilateral assisted gait modalities (C, 25% and 50%) for each subject.

## Abbreviations

SD: standard deviation
NG: normal gait
UAG: unilateral assisted gait
C: comfortable
25%: 25% of the subject body weight
50%: 50% of the subject body weight or the maximum load the subject was able of applying
Il: ipsilateral
Cl: contralateral
CI: confidence interval
Ns: not significant
