# Supplementary material for: Influence of the load exerted over a forearm crutch in spatiotemporal step parameters during assisted gait: pilot study
Source: Biomed Eng Online. 2018 Jul 18;17:98. doi: 10.1186/s12938-018-0527-z (PMC6052579; doi:10.1186/s12938-018-0527-z)
Supplement: Supplementary file 1 — Additional file 1. Descriptive analysis of study variables. [file 12938_2018_527_MOESM1_ESM.docx]

**Additional File 1 Descriptive analysis of study variables**

|  |  | Mean (SD) | Median |  |  | Mean (SD) | Median |
| --- | --- | --- | --- | --- | --- | --- | --- |
| **Ipsilateral step length**  (m) | NG | 0.64(0.05) | 0.62 | **Contralateral step length**  (m) | NG | 0.64(0.05) | 0.62 |
|  | C | 0.63(0.06) | 0.64 |  | C | 0.59(0.07) | 0.60 |
|  | 25% | 0.60(0.08) | 0.61 |  | 25% | 0.58(0.08) | 0.57 |
|  | 50% | 0.59(0.10) | 0.59 |  | 50% | 0.55(0.10) | 0.54 |
| **Ipsilateral step period**  (s) | NG | 0.59(0.04) | 0.58 | **Contralateral step period**  (s) | NG | 0.59(0.04) | 0.58 |
|  | C | 0.71(0.08) | 0.70 |  | C | 0.69(0.09) | 0.67 |
|  | 25% | 0.78(0.15) | 0.73 |  | 25% | 0.73(0.19) | 0.69 |
|  | 50% | 0.86(0.19) | 0.83 |  | 50% | 0.82(0.19) | 0.77 |
| **Velocity**  (m/s) | NG | 1.09(0.11) | 1.07 | **Step width**  (m) | NG | 0.09(0.04) | 0.08 |
|  | C | 0.91(0.15) | 0.95 |  | C | 1.00(5.40) | 0.07 |
|  | 25% | 0.81(0.20) | 0.87 |  | 25% | 0.85(5.03) | 0.09 |
|  | 50% | 0.73(0.21) | 0.70 |  | 50% | 0.80(4.66) | 0.09 |
| **Ipsilateral step angle**  (degrees) | NG | 23.27(6.84) | 22.94 | **Contralateral step angle**  (degrees) | NG | 23.27(6.84) | 22.94 |
|  | C | 31.32(5.48) | 20.32 |  | C | 25.44(3.95) | 25.04 |
|  | 25% | 31.39(5.30) | 20.99 |  | 25% | 24.54(4.57) | 24.61 |
|  | 50% | 21.10(5.71) | 20.22 |  | 50% | 23.76(5.31) | 23.20 |

NG, normal gait; C, assisted gait in which a comfortable load is applied.
