# Supplementary material for: Influence of the load exerted over a forearm crutch in spatiotemporal step parameters during assisted gait: pilot study
Source: Biomed Eng Online. 2018 Jul 18;17:98. doi: 10.1186/s12938-018-0527-z (PMC6052579; doi:10.1186/s12938-018-0527-z)
Supplement: Supplementary file 2 — Additional file 2. Descriptive analysis of step length. [file 12938_2018_527_MOESM2_ESM.docx]

**Additional File 2 Descriptive analysis of step length**

| **STEP LENGTH (m)** | | | | | | | | | | | | |
| --- | --- | --- | --- | --- | --- | --- | --- | --- | --- | --- | --- | --- |
|  | **Ipsilateral step length** | | | | | | **Contralateral step length** | | | | | |
| **Subject** |  | Mean (SD) | Min/Max | Percentiles | | |  | Mean (SD) | Min/Max | Percentiles | | |
|  |  |  |  | 25 | 50 | 75 |  |  |  | 25 | 50 | 75 |
| **1** | NG | 0.66(0.04) | 0.61/0.71 | 0.63 | 0.66 | 0.67 | NG | 0.66(0.04) | 0.56/0.75 | 0.64 | 0.66 | 0.69 |
|  | C | 0.57(0.04) | 0.53/0.62 | 0.54 | 0.57 | 0.61 | C | 0.58(0.02) | 0.55/0.61 | 0.57 | 0.58 | 0.60 |
|  | 25% | 0.53(0.04) | 0.43/0.56 | 0.53 | 0.54 | 0.56 | 25% | 0.52(0.02) | 0.47/0.55 | 0.51 | 0.53 | 0.54 |
|  | 50% | 0.50(0.02) | 0.46/0.53 | 0.48 | 0.50 | 0.51 | 50% | 0.48(0.03) | 0.44/0.55 | 0.46 | 0.48 | 0.51 |
| **2** | NG | 0.62(0.02) | 0.59/0.66 | 0.61 | 0.62 | 0.63 | NG | 0.60(0.02) | 0.57/0.64 | 0.58 | 0.60 | 0.62 |
|  | C | 0.61(0.04) | 0.53/0.68 | 0.57 | 0.60 | 0.64 | C | 0.56(0.02) | 0.53/0.59 | 0.54 | 0.56 | 0.58 |
|  | 25% | 0.59(0.04) | 0.52/0.65 | 0.57 | 0.59 | 0.63 | 25% | 0.53(0.03) | 0.49/0.59 | 0.51 | 0.54 | 0.55 |
|  | 50% | 0.51(0.03) | 0.46/0.57 | 0.49 | 0.50 | 0.52 | 50% | 0.49(0.03) | 0.44/0.52 | 0.47 | 0.49 | 0.51 |
| **3** | NG | 0.61 (0.01) | 0.59/0.62 | 0.60 | 0.61 | 0.62 | NG | 0.61(0.02) | 0.59/0.65 | 0.60 | 0.61 | 0.62 |
|  | C | 0.68(0.01) | 0.67/0.69 | 0.68 | 0.68 | 0.68 | C | 0.66(0.02) | 0.63/0.70 | 0.64 | 0.65 | 0.65 |
|  | 25% | 0.68(0.02) | 0.66/0.72 | 0.68 | 0.68 | 0.68 | 25% | 0.69(0.01) | 0.67/0.72 | 0.68 | 0.69 | 0.69 |
|  | 50% | 0.69(0.04) | 0.61/0.77 | 0.68 | 0.69 | 0.70 | 50% | 0.67(0.02) | 0.64/0.72 | 0.66 | 0.67 | 0.67 |
| **4** | NG | 0.60(0.02) | 0.57/0.64 | 0.57 | 0.59 | 0.59 | NG | 0.60(0.02) | 0.59/0.63 | 0.60 | 0.61 | 0.62 |
|  | C | 0.61(0.01) | 0.60/0.62 | 0.61 | 0.61 | 0.61 | C | 0.60(0.00) | 0.60/0.61 | 0.60 | 0.60 | 0.60 |
|  | 25% | 0.50(0.03) | 0.42/0.53 | 0.49 | 0.52 | 0.53 | 25% | 0.52(0.02) | 0.49/0.55 | 0.51 | 0.52 | 0.54 |
|  | 50% | 0.50(0.056 | 0.38/0.57 | 0.46 | 0.51 | 0.56 | 50% | 0.51(0.05) | 0.45/0.59 | 0.48 | 0.50 | 0.54 |
| **5** | NG | 0.68(0.02) | 0.65/0.71 | 0.66 | 0.68 | 0.69 | NG | 0.74(0.04) | 0.69/0.81 | 0.71 | 0.74 | 0.76 |
|  | C | 0.65(0.02) | 0.62/0.69 | 0.63 | 0.65 | 0.66 | C | 0.68(0.03) | 0.65/0.77 | 0.66 | 0.68 | 0.68 |
|  | 25% | 0.52(0.02) | 0.48/0.56 | 0.51 | 0.52 | 0.54 | 25% | 0.65(0.04) | 0.59/0.69 | 0.63 | 0.65 | 0.68 |
|  | 50% | 0.55(0.04) | 0.48/0.61 | 0.52 | 0.55 | 0.57 | 50% | 0.61(0.04) | 0.56/0.67 | 0.57 | 0.62 | 0.65 |
| **6** | NG | 0.62(0.03) | 0.59/0.64 | 0.60 | 0.61 | 0.62 | NG | 0.62(0.03) | 0.59/0.68 | 0.61 | 0.63 | 0.64 |
|  | C | 0.69(0.02) | 0.65/0.71 | 0.67 | 0.68 | 0.71 | C | 0.63(0.02) | 0.58/0.64 | 0.61 | 0.63 | 0.64 |
|  | 25% | 0.69(0.02) | 0.66/0.73 | 0.67 | 0.68 | 0.71 | 25% | 0.62(0.02) | 0.60/0.64 | 0.60 | 0.63 | 0.63 |
|  | 50% | 0.69(0.03) | 0.65/0.73 | 0.67 | 0.68 | 0.71 | 50% | 0.63(0.02) | 0.61/0.68 | 0.62 | 0.63 | 0.64 |
| **7** | NG | 0.62(0.00) | 0.62/0.62 | 0.62 | 0.62 | 0.62 | NG | 0.68(0.00) | 0.68/0.68 | 0.68 | 0.68 | 0.68 |
|  | C | 0.70(0.04) | 0.66/0.80 | 0.67 | 0.69 | 0.70 | C | 0.59(0.11) | 0.29/0.65 | 0.59 | 0.63 | 0.65 |
|  | 25% | 0.69(0.03) | 0.63/0.75 | 0.69 | 0.69 | 0.70 | 25% | 0.66(0.01) | 0.64/0.68 | 0.66 | 0.66 | 0.67 |
|  | 50% | 0.70(0.03) | 0.66/0.79 | 0.69 | 0.70 | 0.70 | 50% | 0.69(0.02) | 0.67/0.73 | 0.68 | 0.69 | 0.70 |
| **8** | NG | 0.61(0.02) | 0.59/0.64 | 0.60 | 0.61 | 0.62 | NG | 0.63(0.03) | 0.59/0.68 | 0.61 | 0.63 | 0.64 |
|  | C | 0.61(0.03) | 0.57/0.67 | 0.58 | 0.61 | 0.64 | C | 0.59(0.03) | 0.54/0.62 | 0.56 | 0.58 | 0.62 |
|  | 25% | 0.63(0.04) | 0.57/0.71 | 0.60 | 0.62 | 0.64 | 25% | 0.58(0.03) | 0.54/0.63 | 0.57 | 0.58 | 0.61 |
|  | 50% | 0.62(0.02) | 0.59/0.65 | 0.60 | 0.62 | 0.63 | 50% | 0.56(0.10) | 0.29/0.65 | 0.57 | 0.58 | 0.61 |
| **9** | NG | 0.59(0.02) | 0.57/0.64 | 0.57 | 0.59 | 0.59 | NG | 0.61(0.01) | 0.59/0.63 | 0.60 | 0.61 | 0.62 |
|  | C | 0.60(0.02) | 0.58/0.64 | 0.60 | 0.60 | 0.61 | C | 0.50(0.07) | 0.33/0.56 | 0.48 | 0.52 | 0.55 |
|  | 25% | 0.58(0.03) | 0.53/0.63 | 0.56 | 0.58 | 0.61 | 25% | 0.54(0.04) | 0.46/0.62 | 0.51 | 0.54 | 0.55 |
|  | 50% | 0.56(0.04) | 0.52/0.63 | 0.53 | 0.56 | 0.59 | 50% | 0.48(0.13) | 0.23/0.58 | 0.44 | 0.52 | 0.55 |
| **10** | NG | 0.71(0.03) | 0.67/0.73 | 0.67 | 0.70 | 0.71 | NG | 0.71(0.03) | 0.69/0.81 | 0.69 | 0.70 | 0.72 |
|  | C | 0.64(0.01) | 0.62/0.67 | 0.63 | 0.64 | 0.64 | C | 0.60(0.02) | 0.58/0.64 | 0.59 | 0.60 | 0.60 |
|  | 25% | 0.64(0.03) | 0.59/0.67 | 0.61 | 0.65 | 0.67 | 25% | 0.56(0.02) | 0.51/0.59 | 0.54 | 0.57 | 0.57 |
|  | 50% | 0.66(0.02) | 0.63/0.71 | 0.64 | 0.66 | 0.67 | 50% | 0.53(0.03) | 0.48/0.58 | 0.51 | 0.53 | 0.54 |
| **11** | NG | 0.66(0.03) | 0.61/0.71 | 0.63 | 0.66 | 0.67 | NG | 0.66(0.05) | 0.56/0.75 | 0.64 | 0.66 | 0.69 |
|  | C | 0.53(0.03) | 0.47/0.57 | 0.51 | 0.53 | 0.55 | C | 0.50(0.03) | 0.44/0.54 | 0.47 | 0.50 | 0.53 |
|  | 25% | 0.49(0.05) | 0.44/0.58 | 0.45 | 0.48 | 0.51 | 25% | 0.47(0.03) | 0.43/0.52 | 0.45 | 0.47 | 0.50 |
|  | 50% | 0.43(0.03) | 0.38/0.46 | 0.42 | 0.43 | 0.46 | 50% | 0.42(0.04) | 0.33/0.50 | 0.39 | 0.42 | 0.44 |

N=10. NG, normal gait; C, assisted gait in which a comfortable load is applied; 25%, assisted gait in which a 25% of body weight bearing is applied; 50%, assisted gait in which a 50% of body weight bearing is applied.
