# Supplementary material for: Influence of the load exerted over a forearm crutch in spatiotemporal step parameters during assisted gait: pilot study
Source: Biomed Eng Online. 2018 Jul 18;17:98. doi: 10.1186/s12938-018-0527-z (PMC6052579; doi:10.1186/s12938-018-0527-z)
Supplement: Supplementary file 3 — Additional file 3. Descriptive analysis of step period. [file 12938_2018_527_MOESM3_ESM.docx]

**Additional File 3 Descriptive analysis of step period**

| **STEP PERIOD (s)** | | | | | | | | | | | | |
| --- | --- | --- | --- | --- | --- | --- | --- | --- | --- | --- | --- | --- |
|  | **Ipsilateral step period** | | | | | | **Contralateral step period** | | | | | |
| **Subject** |  | Mean (SD) | Min/Max | Percentiles | | |  | Mean (SD) | Min/Max | Percentiles | | |
|  |  |  |  | 25 | 50 | 75 |  |  |  | 25 | 50 | 75 |
| **1** | NG | 0.62(0.03) | 0.55/0.65 | 0.60 | 0.61 | 0.64 | NG | 0.62(0.03) | 0.58/0.65 | 0.60 | 0.62 | 0.63 |
|  | C | 0.76(0.02) | 0.72/0.80 | 0.74 | 0.76 | 0.78 | C | 0.78(0.04) | 0.73/0.83 | 0.74 | 0.77 | 0.81 |
|  | 25% | 1.02(0.05) | 0.94/1.09 | 0.97 | 1.04 | 1.06 | 25% | 1.00(0.06) | 0.88/1.12 | 0.97 | 0.99 | 1.02 |
|  | 50% | 1.10(0.08) | 0.95/1.21 | 1.03 | 1.12 | 1.16 | 50% | 1.06(0.08) | 0.96/1.23 | 1.01 | 1.04 | 1.13 |
| **2** | NG | 0.61(0.03) | 0.57/0.65 | 0.58 | 0.60 | 0.63 | NG | 0.61(0.03) | 0.57/0.65 | 0.58 | 0.60 | 0.63 |
|  | C | 0.74(0.03) | 0.70/0.77 | 0.72 | 0.75 | 0.76 | C | 0.74(0.03) | 0.70/0.77 | 0.72 | 0.75 | 0.76 |
|  | 25% | 0.85(0.04) | 0.79/0.92 | 0.81 | 0.85 | 0.87 | 25% | 0.85(0.04) | 0.79/0.92 | 0.81 | 0.85 | 0.87 |
|  | 50% | 0.93(0.07) | 0.84/1.04 | 0.85 | 0.94 | 0.98 | 50% | 0.93(0.07) | 0.84/1.04 | 0.85 | 0.94 | 0.98 |
| **3** | NG | 0.57(0.01) | 0.55/0.58 | 0.56 | 0.57 | 0.57 | NG | 0.57(0.01) | 0.55/0.59 | 0.56 | 0.57 | 0.58 |
|  | C | 0.63(0.00) | 0.62/0.63 | 0.63 | 0.63 | 0.63 | C | 0.60(0.02) | 0.57/0.65 | 0.60 | 0.60 | 0.60 |
|  | 25% | 0.62(0.01) | 0.61/0.63 | 0.62 | 0.62 | 0.62 | 25% | 0.62(0.02) | 0.59/0.67 | 0.62 | 0.62 | 0.62 |
|  | 50% | 0.72(0.03) | 0.65/0.77 | 0.71 | 0.72 | 0.73 | 50% | 0.70(0.03) | 0.67/0.77 | 0.68 | 0.70 | 0.70 |
| **4** | NG | 0.62(0.04) | 0.55/0.71 | 0.58 | 0.61 | 0.63 | NG | 0.62(0.04) | 0.60/0.75 | 0.61 | 0.62 | 0.63 |
|  | C | 0.68(0.01) | 0.65/0.71 | 0.68 | 0.68 | 0.68 | C | 0.67(0.02) | 0.62/0.72 | 0.67 | 0.67 | 0.67 |
|  | 25% | 0.84(0.06) | 0.73/0.96 | 0.80 | 0.84 | 0.86 | 25% | 0.87(0.03) | 0.83/0.94 | 0.85 | 0.86 | 0.89 |
|  | 50% | 0.92(0.09) | 0.69/1.00 | 0.90 | 0.93 | 0.96 | 50% | 0.93(0.07) | 0.83/1.06 | 0.86 | 0.92 | 0.98 |
| **5** | NG | 0.53(0.02) | 0.50/0.55 | 0.52 | 0.53 | 0.55 | NG | 0.58(0.02) | 0.55/0.61 | 0.56 | 0.58 | 0.59 |
|  | C | 0.60(0.02) | 0.55/0.62 | 0.59 | 0.60 | 0.61 | C | 0.63(0.03) | 0.58/0.67 | 0.61 | 0.63 | 0.64 |
|  | 25% | 0.59(0.03) | 0.55/0.64 | 0.57 | 0.59 | 0.62 | 25% | 0.74(0.04) | 0.68/0.79 | 0.72 | 0.74 | 0.77 |
|  | 50% | 0.59(0.04) | 0.53/0.68 | 0.56 | 0.58 | 0.60 | 50% | 0.66(0.05) | 0.60/0.72 | 0.62 | 0.66 | 0.70 |
| **6** | NG | 0.59(0.03) | 0.50/0.63 | 0.57 | 0.58 | 0.61 | NG | 0.59(0.03) | 0.57/0.63 | 0.59 | 0.60 | 0.60 |
|  | C | 0.71(0.02) | 0.68/0.74 | 0.70 | 0.70 | 0.70 | C | 0.65(0.01) | 0.62/0.67 | 0.65 | 0.65 | 0.65 |
|  | 25% | 0.73(0.02) | 0.69/0.78 | 0.71 | 0.72 | 0.73 | 25% | 0.66(0.02) | 0.62/0.70 | 0.64 | 0.66 | 0.68 |
|  | 50% | 0.78(0.01) | 0.75/0.80 | 0.78 | 0.78 | 0.78 | 50% | 0.70(0.01) | 0.69/0.73 | 0.70 | 0.70 | 0.70 |
| **7** | NG | 0.52(0.00) | 0.52/0.52 | 0.52 | 0.52 | 0.52 | NG | 0.57(0.00) | 0.57/0.57 | 0.57 | 0.57 | 0.57 |
|  | C | 0.69(0.04) | 0.62/0.78 | 0.66 | 0.69 | 0.69 | C | 0.58(0.11) | 0.28/0.69 | 0.58 | 0.59 | 0.64 |
|  | 25% | 0.71(0.02) | 0.67/0.76 | 0.71 | 0.71 | 0.71 | 25% | 0.69(0.01) | 0.67/0.71 | 0.68 | 0.69 | 0.69 |
|  | 50% | 0.73(0.04) | 0.66/0.81 | 0.69 | 0.73 | 0.74 | 50% | 0.72(0.03) | 0.67/0.76 | 0.69 | 0.71 | 0.75 |
| **8** | NG | 0.58(0.04) | 0.50/0.63 | 0.57 | 0.58 | 0.61 | NG | 0.60(0.02) | 0.57/0.63 | 0.59 | 0.60 | 0.60 |
|  | C | 0.69(0.04) | 0.63/0.73 | 0.67 | 0.68 | 0.72 | C | 0.68(0.03) | 0.61/0.71 | 0.66 | 0.69 | 0.70 |
|  | 25% | 0.73(0.04) | 0.66/0.77 | 0.69 | 0.73 | 0.77 | 25% | 0.64(0.14) | 0.28/0.78 | 0.63 | 0.66 | 0.71 |
|  | 50% | 0.76(0.04) | 0.70/0.82 | 0.73 | 0.76 | 0.80 | 50% | 0.73(0.04) | 0.68/0.78 | 0.68 | 0.73 | 0.75 |
| **9** | NG | 0.61(0.04) | 0.55/0.71 | 0.58 | 0.61 | 0.63 | NG | 0.63(0.04) | 0.60/0.75 | 0.61 | 0.62 | 0.63 |
|  | C | 0.76(0.02) | 0.74/0.79 | 0.75 | 0.75 | 0.76 | C | 0.69(0.02) | 0.67/0.72 | 0.68 | 0.69 | 0.71 |
|  | 25% | 0.82(0.03) | 0.77/0.87 | 0.79 | 0.81 | 0.85 | 25% | 0.63(0.17) | 0.35/0.80 | 0.46 | 0.69 | 0.74 |
|  | 50% | 0.82(0.06) | 0.74/0.93 | 0.77 | 0.83 | 0.85 | 50% | 0.74(0.03) | 0.68/0.77 | 0.72 | 0.74 | 0.76 |
| **10** | NG | 0.56(0.02) | 0.50/0.58 | 0.53 | 0.55 | 0.57 | NG | 0.56(0.02) | 0.55/0.61 | 0.56 | 0.58 | 0.59 |
|  | C | 0.63(0.01) | 0.62/0.64 | 0.63 | 0.63 | 0.63 | C | 0.59(0.01) | 0.58/0.61 | 0.59 | 0.59 | 0.59 |
|  | 25% | 0.65(0.03) | 0.61/0.69 | 0.63 | 0.65 | 0.67 | 25% | 0.57(0.02) | 0.52/0.61 | 0.55 | 0.57 | 0.59 |
|  | 50% | 0.68(0.02) | 0.65/0.71 | 0.66 | 0.68 | 0.70 | 50% | 0.54(0.03) | 0.49/0.58 | 0.53 | 0.54 | 0.56 |
| **11** | NG | 0.61(0.03) | 0.55/0.65 | 0.60 | 0.61 | 0.64 | NG | 0.62(0.02) | 0.58/0.65 | 0.60 | 0.62 | 0.63 |
|  | C | 0.84(0.07) | 0.75/0.95 | 0.78 | 0.84 | 0.91 | C | 0.79(0.06) | 0.68/0.86 | 0.75 | 0.79 | 0.84 |
|  | 25% | 1.09(0.07) | 0.96/1.19 | 1.03 | 1.09 | 1.14 | 25% | 1.07(0.13) | 0.89/1.28 | 0.92 | 1.08 | 1.15 |
|  | 50% | 1.28(0.11) | 1.16/1.46 | 1.20 | 1.25 | 1.36 | 50% | 1.23(0.09) | 1.07/1.37 | 1.16 | 1.21 | 1.31 |

N=10. NG, normal gait; C, assisted gait in which a comfortable load is applied; 25%, assisted gait in which a 25% of body weight bearing is applied; 50%, assisted gait in which a 50% of body weight bearing is applied.
