# Supplementary material for: Influence of the load exerted over a forearm crutch in spatiotemporal step parameters during assisted gait: pilot study
Source: Biomed Eng Online. 2018 Jul 18;17:98. doi: 10.1186/s12938-018-0527-z (PMC6052579; doi:10.1186/s12938-018-0527-z)
Supplement: Supplementary file 4 — Additional file 4. Descriptive analysis of velocity. [file 12938_2018_527_MOESM4_ESM.docx]

**Additional File 4 Descriptive analysis of velocity**

| **VELOCITY (m/s)** | | | | | | |
| --- | --- | --- | --- | --- | --- | --- |
| **Subject** |  | Mean (SD) | Min/Max | Percentiles | | |
|  |  |  |  | 25 | 50 | 75 |
| **1** | NG | 1.07(0.07) | 0.97/1.21 | 1.01 | 1.07 | 1.11 |
|  | C | 0.76(0.05) | 0.67/0.82 | 0.71 | 0.76 | 0.80 |
|  | 25% | 0.52(0.03) | 0.45/0.56 | 0.51 | 0.53 | 0.54 |
|  | 50% | 0.46(0.03) | 0.41/0.51 | 0.44 | 0.44 | 0.49 |
| **2** | NG | 1.03(0.03) | 0.98/1.07 | 1.00 | 1.03 | 1.06 |
|  | C | 0.82(0.05) | 0.76/0.91 | 0.77 | 0.81 | 0.86 |
|  | 25% | 0.70(0.05) | 0.65/0.80 | 0.66 | 0.70 | 0.73 |
|  | 50% | 0.55(0.03) | 0.48/0.60 | 0.52 | 0.54 | 0.57 |
| **3** | NG | 1.08(0.03) | 1.05/1.13 | 1.05 | 1.07 | 1.09 |
|  | C | 1.09(0.01) | 1.07/1.11 | 1.08 | 1.09 | 1.09 |
|  | 25% | 1.11(0.02) | 1.07/1.15 | 1.11 | 1.11 | 1.11 |
|  | 50% | 0.96(0.02) | 0.94/1.00 | 0.96 | 0.96 | 0.96 |
| **4** | NG | 0.97(0.06) | 0.80/1.04 | 0.95 | 0.97 | 1.01 |
|  | C | 0.92(0.03) | 0.85/0.96 | 0.90 | 0.90 | 0.90 |
|  | 25% | 0.60(0.03) | 0.55/0.65 | 0.58 | 0.60 | 0.62 |
|  | 50% | 0.55(0.05) | 0.45/0.62 | 0.51 | 0.56 | 0.58 |
| **5** | NG | 1.27(0.03) | 1.23/1.32 | 1.25 | 1.27 | 1.31 |
|  | C | 1.09(0.03) | 1.03/1.14 | 1.08 | 1.09 | 1.11 |
|  | 25% | 0.88(0.02) | 0.85/0.92 | 0.87 | 0.88 | 0.89 |
|  | 50% | 0.93(0.02) | 0.90/0.97 | 0.91 | 0.94 | 0.95 |
| **6** | NG | 1.05(0.07) | 0.95/1.21 | 1.01 | 1.05 | 1.06 |
|  | C | 0.97(0.01) | 0.95/0.98 | 0.97 | 0.97 | 0.97 |
|  | 25% | 0.92(0.03) | 0.87/0.97 | 0.92 | 0.92 | 0.93 |
|  | 50% | 0.91(0.01) | 0.88/0.93 | 0.91 | 0.91 | 0.91 |
| **7** | NG | 1.18(0.00) | 1.18/1.18 | 1.18 | 1.18 | 1.18 |
|  | C | 1.02(0.04) | 0.94/1.11 | 1.01 | 1.02 | 1.03 |
|  | 25% | 0.97(0.01) | 0.94/0.99 | 0.97 | 0.97 | 0.97 |
|  | 50% | 0.97(0.04) | 0.91/1.04 | 0.95 | 0.97 | 0.99 |
| **8** | NG | 1.05(0.07) | 0.95/1.21 | 1.01 | 1.05 | 1.06 |
|  | C | 0.89(0.06) | 0.80/0.99 | 0.84 | 0.89 | 0.93 |
|  | 25% | 0.87(0.10) | 0.74/1.03 | 0.80 | 0.87 | 0.92 |
|  | 50% | 0.81(0.03) | 0.77/0.85 | 0.78 | 0.81 | 0.84 |
| **9** | NG | 0.97(0.07) | 0.81/1.04 | 0.95 | 0.97 | 1.01 |
|  | C | 0.80(0.03) | 0.74/0.86 | 0.79 | 0.80 | 0.81 |
|  | 25% | 0.71(0.05) | 0.66/0.81 | 0.67 | 0.69 | 0.76 |
|  | 50% | 0.69(0.05) | 0.61/0.75 | 0.64 | 0.70 | 0.74 |
| **10** | NG | 1.27(0.03) | 1.23/1.32 | 1.25 | 1.27 | 1.31 |
|  | C | 1.01(0.02) | 0.98/1.06 | 1.01 | 1.01 | 1.02 |
|  | 25% | 0.99(0.04) | 0.94/1.06 | 0.96 | 0.97 | 1.00 |
|  | 50% | 0.98(0.02) | 0.95/1.00 | 0.96 | 0.98 | 0.99 |
| **11** | NG | 1.07(0.07) | 0.97/1.20 | 1.01 | 1.07 | 1.11 |
|  | C | 0.63(0.04) | 0.58/0.74 | 0.61 | 0.62 | 0.64 |
|  | 25% | 0.45(0.05) | 0.40/0.57 | 0.41 | 0.43 | 0.48 |
|  | 50% | 0.34(0.02) | 0.31/0.36 | 0.32 | 0.34 | 0.36 |

N=10. NG, normal gait; C, assisted gait in which a comfortable load is applied; 25%, assisted gait in which a 25% of body weight bearing is applied; 50%, assisted gait in which a 50% of body weight bearing is applied.
