# Supplementary material for: Influence of the load exerted over a forearm crutch in spatiotemporal step parameters during assisted gait: pilot study
Source: Biomed Eng Online. 2018 Jul 18;17:98. doi: 10.1186/s12938-018-0527-z (PMC6052579; doi:10.1186/s12938-018-0527-z)
Supplement: Supplementary file 5 — Additional file 5. Descriptive analysis of step angle. [file 12938_2018_527_MOESM5_ESM.docx]

**Additional File 5 Descriptive analysis of step angle**

| **STEP ANGLE (degrees)** | | | | | | | | | | | | |
| --- | --- | --- | --- | --- | --- | --- | --- | --- | --- | --- | --- | --- |
|  | **Ipsilateral step angle** | | | | | | **Contralateral step angle** | | | | | |
| **Subject** |  | Mean (SD) | Min/Max | Percentiles | | |  | Mean (SD) | Min/Max | Percentiles | | |
|  |  |  |  | 25 | 50 | 75 |  |  |  | 25 | 50 | 75 |
| **1** | NG | 28.89(3.65) | 26.13/35.62 | 28.72 | 31.10 | 32.16 | NG | 28.89(3.65) | 21.59/34.95 | 22.82 | 26.36 | 31.12 |
|  | C | 21.84(3.92) | 16.59/27.85 | 18.43 | 21.93 | 24.94 | C | 26.22(1.41) | 23.23/27.58 | 25.15 | 26.56 | 27.36 |
|  | 25% | 22.23(5.26) | 14.92/29.63 | 15.77 | 23.61 | 26.36 | 25% | 26.57(2.23) | 23.61/29.38 | 24.47 | 26.45 | 28.70 |
|  | 50% | 21.74(3.81) | 16.36/28.28 | 18.23 | 21.25 | 25.04 | 50% | 25.63(2.20) | 22.26/29.86 | 24.16 | 25.49 | 26.76 |
| **2** | NG | 32.12(3.62) | 24.20/37.34 | 31.11 | 31.47 | 35.07 | NG | 19.06(3.88) | 15.38/29.40 | 17.15 | 18.01 | 19.54 |
|  | C | 19.64(2.98) | 15.05/24.99 | 17.48 | 19.32 | 22.40 | C | 25.59(2.30) | 20.84/28.21 | 24.30 | 26.20 | 27.51 |
|  | 25% | 22.01(2.61) | 17.51/25.53 | 19.97 | 22.60 | 24.02 | 25% | 21.45(3.25) | 15.16/26.05 | 19.40 | 21.72 | 23.96 |
|  | 50% | 22.71(3.13) | 16.15/26.51 | 20.67 | 23.17 | 25.35 | 50% | 17.74(2.18) | 14.29/21.08 | 16.04 | 17.98 | 19.15 |
| **3** | NG | 29.99(2.77) | 26.02/35.17 | 27.82 | 30.13 | 31.28 | NG | 22.57(3.86) | 16.48/29.75 | 20.00 | 22.61 | 25.20 |
|  | C | 30.66(2.04) | 25.63/33.88 | 30.66 | 30.66 | 31.53 | C | 31.64(0.75) | 29.95/33.00 | 31.59 | 31.64 | 31.78 |
|  | 25% | 30.34(1.13) | 27.97/32.76 | 30.33 | 30.34 | 30.34 | 25% | 31.39(0.49) | 30.21/32.12 | 31.39 | 31.39 | 31.51 |
|  | 50% | 29.35(3.40) | 22.83/35.60 | 27.93 | 29.35 | 30.30 | 50% | 33.20(4.98) | 24.81/45.20 | 32.11 | 33.20 | 33.20 |
| **4** | NG | 18.24(2.66) | 10.88/25.54 | 16.05 | 18.43 | 22.35 | NG | 18.24(2.66) | 13.16/22.99 | 16.35 | 17.28 | 18.67 |
|  | C | 16.49(0.94) | 14.50/18.47 | 16.49 | 16.49 | 16.49 | C | 24.11(0.53) | 22.99/25.24 | 24.11 | 24.11 | 24.11 |
|  | 25% | 15.55(2.26) | 12.92/19.74 | 13.84 | 14.86 | 17.51 | 25% | 25.00(1.72) | 22.99/27.77 | 23.61 | 24.33 | 26.72 |
|  | 50% | 16.63(2.90) | 13.13/21.00 | 13.61 | 16.40 | 19.51 | 50% | 24.47(2.33) | 21.36/28.55 | 22.85 | 23.80 | 27.00 |
| **5** | NG | 28.55(3.11) | 22.66/31.97 | 25.69 | 29.10 | 31.32 | NG | 25.10(3.50) | 19.49/31.45 | 22.31 | 25.10 | 27.31 |
|  | C | 26.85(4.70) | 19.96/33.34 | 22.08 | 24.830 | 31.23 | C | 24.41(1.34) | 22.50/26.62 | 23.42 | 24.41 | 25.00 |
|  | 25% | 20.65(4.47) | 14.55/27.47 | 16.51 | 21.02 | 24.18 | 25% | 24.51(1.70) | 21.70/27.18 | 23.17 | 24.59 | 25.99 |
|  | 50% | 23.71(5.20) | 16.24/31.45 | 18.68 | 24.32 | 26.73 | 50% | 22.05(1.83) | 18.05/24.19 | 20.47 | 22.75 | 23.15 |
| **6** | NG | 20.87(3.25) | 17.41/30.63 | 17.30 | 18.13 | 21.03 | NG | 20.87(3.25) | 12.42/26.15 | 15.54 | 18.64 | 22.35 |
|  | C | 24.90(2.93) | 17.76/28.89 | 24.89 | 24.90 | 25.69 | C | 26.37(2.05) | 23.15/30.51 | 25.01 | 26.89 | 27.11 |
|  | 25% | 21.57(5.23) | 8.87/26.70 | 20.52 | 21.57 | 25.43 | 25% | 26.78(2.22) | 22.35/30.95 | 25.39 | 27.00 | 27.86 |
|  | 50% | 20.22(3.47) | 14.49/25.52 | 19.57 | 20.22 | 20.22 | 50% | 26.34(1.98) | 21.28/28.88 | 25.97 | 26.89 | 27.12 |
| **7** | NG | 21.95(0.00) | 21.95/21.95 | 21.95 | 21.95 | 21.95 | NG | 10.44(0.00) | 10.44/10.44 | 10.44 | 10.44 | 10.44 |
|  | C | 20.32(3.44) | 15.20/28.23 | 18.60 | 20.32 | 21.52 | C | 19.86(2.45) | 13.79/22.42 | 19.44 | 19.86 | 21.72 |
|  | 25% | 21.42(1.33) | 19.19/24.62 | 21.10 | 21.42 | 21.42 | 25% | 16.50(2.46) | 11.95/21.63 | 15.95 | 16.50 | 16.91 |
|  | 50% | 20.47(2.58) | 15.82/24.02 | 18.74 | 20.47 | 22.46 | 50% | 19.22(6.40) | 1.81/23.46 | 19.13 | 20.25 | 23.11 |
| **8** | NG | 25.13(2.68) | 21.51/30.63 | 23.30 | 25.13 | 26.03 | NG | 16.60(3.82) | 12.42/26.15 | 13.54 | 16.51 | 17.04 |
|  | C | 22.53(4.18) | 17.73/29.97 | 19.66 | 21.81 | 25.02 | C | 23.85(2.28) | 20.18/27.99 | 22.30 | 23.71 | 25.18 |
|  | 25% | 18.64(5.02) | 10.42/27.80 | 15.00 | 18.25 | 21.73 | 25% | 22.79(1.78) | 18.46/25.57 | 22.60 | 22.79 | 23.69 |
|  | 50% | 18.42(4.34) | 11.47/25.90 | 14.59 | 19.09 | 21.04 | 50% | 22.14(0.82) | 20.10/23.36 | 22.12 | 22.16 | 22.39 |
| **9** | NG | 21.04(4.01) | 10.88/25.54 | 20.43 | 21.99 | 23.12 | NG | 15.43(1.30) | 13.16/17.99 | 14.55 | 15.51 | 15.92 |
|  | C | 18.99(1.20) | 16.40/20.22 | 18.54 | 18.99 | 20.06 | C | 20.30(1.25) | 18.15/22.69 | 19.51 | 20.30 | 20.83 |
|  | 25% | 18.98(2.29) | 15.85/22.88 | 17.08 | 18.56 | 20.65 | 25% | 18.78(1.41) | 16.16/20.68 | 18.04 | 18.55 | 20.14 |
|  | 50% | 18.62(3.48) | 10.50/22.47 | 16.83 | 18.84 | 21.09 | 50% | 18.33(2.22) | 14.83/21.23 | 16.64 | 18.19 | 20.55 |
| **10** | NG | 26.83(3.31) | 22.66/31.97 | 25.69 | 29.10 | 31.32 | NG | 26.83(3.31) | 19.49/31.45 | 22.31 | 25.10 | 27.31 |
|  | C | 19.72(1.38) | 17.90/22.54 | 18.31 | 19.72 | 20.65 | C | 25.56(2.20) | 22.99/29.96 | 23.97 | 25.10 | 26.95 |
|  | 25% | 18.04(2.35) | 14.97/23.49 | 16.39 | 17.83 | 19.31 | 25% | 26.07(1.98) | 23.49/30.01 | 24.55 | 25.48 | 27.43 |
|  | 50% | 19.63(2.49) | 16.65/25.38 | 17.58 | 19.63 | 20.36 | 50% | 26.14(2.03) | 22.66/28.94 | 24.54 | 25.82 | 28.24 |
| **11** | NG | 30.66(2.70) | 26.13/35.62 | 28.72 | 31.10 | 32.16 | NG | 27.12(4.60) | 21.59/34.95 | 22.82 | 26.36 | 31.12 |
|  | C | 18.19(2.63) | 13.62/21.47 | 15.66 | 18.27 | 20.85 | C | 23.82(2.54) | 18.55/27.58 | 22.39 | 24.42 | 25.45 |
|  | 25% | 18.55(3.44) | 13.13/25.46 | 16.27 | 18.65 | 20.29 | 25% | 24.10(3.72) | 18.27/29.76 | 21.26 | 23.47 | 27.76 |
|  | 50% | 19.46(3.67) | 15.48/27.31 | 16.27 | 18.92 | 21.47 | 50% | 19.40(2.64) | 15.67/23.75 | 17.25 | 18.56 | 22.27 |

N=10. NG, normal gait; C, assisted gait in which a comfortable load is applied; 25%, assisted gait in which a 25% of body weight bearing is applied; 50%, assisted gait in which a 50% of body weight bearing is applied.
