# Supplementary material for: Influence of the load exerted over a forearm crutch in spatiotemporal step parameters during assisted gait: pilot study
Source: Biomed Eng Online. 2018 Jul 18;17:98. doi: 10.1186/s12938-018-0527-z (PMC6052579; doi:10.1186/s12938-018-0527-z)
Supplement: Supplementary file 6 — Additional file 6. Descriptive analysis of step width. [file 12938_2018_527_MOESM6_ESM.docx]

**Additional File 6 Descriptive analysis of step width**

| **STEP WIDTH (m)** | | | | | | |
| --- | --- | --- | --- | --- | --- | --- |
| **Subject** |  | Mean (SD) | Min/Max | Percentiles | | |
|  |  |  |  | 25 | 50 | 75 |
| **1** | NG | 0.12(0.03) | 0.08/0.16 | 0.09 | 0.10 | 0.15 |
|  | C | 0.12(0.02) | 0.09/0.15 | 0.10 | 0.13 | 0.14 |
|  | 25% | 0.12(0.02) | 0.09/0.15 | 0.10 | 0.12 | 0.13 |
|  | 50% | 0.10(0.03) | 0.06/0.13 | 0.08 | 0.11 | 0.12 |
| **2** | NG | 0.08(0.01) | 0.06/0.10 | 0.07 | 0.08 | 0.09 |
|  | C | 0.10(0.02) | 0.07/0.12 | 0.08 | 0.10 | 0.11 |
|  | 25% | 0.12(0.08) | 0.07/0.36 | 0.09 | 0.10 | 0.11 |
|  | 50% | 0.11(0.01) | 0.09/0.13 | 0.10 | 0.11 | 0.12 |
| **3** | NG | 0.10(0.10) | 0.02/0.35 | 0.03 | 0.09 | 0.13 |
|  | C | 0.04(0.01) | 0.03/0.05 | 0.04 | 0.04 | 0.04 |
|  | 25% | 0.07(0.00) | 0.07/0.07 | 0.07 | 0.07 | 0.07 |
|  | 50% | 0.05(0.01) | 0.03/0.07 | 0.05 | 0.05 | 0.06 |
| **4** | NG | 0.06(0.02) | 0.04/0.11 | 0.05 | 0.05 | 0.06 |
|  | C | 0.06(0.00) | 0.06/0.06 | 0.06 | 0.06 | 0.06 |
|  | 25% | 0.09(0.01) | 0.08/0.10 | 0.08 | 0.09 | 0.10 |
|  | 50% | 0.11(0.02) | 0.09/0.14 | 0.09 | 0.11 | 0.12 |
| **5** | NG | 0.08(0.03) | 0.04/0.14 | 0.07 | 0.08 | 0.10 |
|  | C | 0.05(0.01) | 0.04/0.08 | 0.04 | 0.05 | 0.05 |
|  | 25% | 0.05(0.02) | 0.02/0.07 | 0.04 | 0.05 | 0.06 |
|  | 50% | 0.06(0.01) | 0.05/0.08 | 0.05 | 0.06 | 0.07 |
| **6** | NG | 0.09(0.02) | 0.06/0.13 | 0.08 | 0.09 | 0.11 |
|  | C | 0.09(0.01) | 0.08/0.10 | 0.09 | 0.09 | 0.09 |
|  | 25% | 0.10(0.01) | 0.08/0.11 | 0.09 | 0.10 | 0.11 |
|  | 50% | 0.09(0.01) | 0.06/0.12 | 0.09 | 0.09 | 0.09 |
| **7** | NG | 0.07(0.00) | 0.07/0.07 | 0.07 | 0.07 | 0.07 |
|  | C | 0.06(0.03) | 0.00/0.11 | 0.04 | 0.06 | 0.09 |
|  | 25% | 0.03(0.01) | 0.02/0.04 | 0.03 | 0.03 | 0.03 |
|  | 50% | 0.06(0.02) | 0.02/0.09 | 0.04 | 0.06 | 0.07 |
| **8** | NG | 0.09(0.02) | 0.06/0.13 | 0.08 | 0.09 | 0.11 |
|  | C | 0.09(0.02) | 0.07/0.12 | 0.07 | 0.07 | 0.10 |
|  | 25% | 0.08(0.01) | 0.05/0.11 | 0.08 | 0.08 | 0.09 |
|  | 50% | 0.08(0.01) | 0.07/0.11 | 0.07 | 0.08 | 0.09 |
| **9** | NG | 0.06(0.02) | 0.04/0.11 | 0.05 | 0.05 | 0.06 |
|  | C | 0.06(0.01) | 0.03/0.07 | 0.06 | 0.06 | 0.06 |
|  | 25% | 0.06(0.02) | 0.03/0.09 | 0.04 | 0.06 | 0.07 |
|  | 50% | 0.08(0.01) | 0.06/0.10 | 0.07 | 0.07 | 0.08 |
| **10** | NG | 0.08(0.03) | 0.04/0.14 | 0.07 | 0.08 | 0.10 |
|  | C | 0.08(0.02) | 0.03/1.00 | 0.07 | 0.08 | 0.09 |
|  | 25% | 0.08(0.02) | 0.04/0.11 | 0.06 | 0.08 | 0.09 |
|  | 50% | 0.07(0.01) | 0.05/0.09 | 0.06 | 0.07 | 0.08 |
| **11** | NG | 0.12(0.03) | 0.08/0.16 | 0.09 | 0.10 | 0.15 |
|  | C | 0.12(0.02) | 0.08/0.14 | 0.11 | 0.12 | 0.13 |
|  | 25% | 0.10(0.01) | 0.08/0.12 | 0.09 | 0.10 | 0.11 |
|  | 50% | 0.12(0.01) | 0.10/0.14 | 0.11 | 0.13 | 0.13 |

N=10. NG, normal gait; C, assisted gait in which a comfortable load is applied; 25%, assisted gait in which a 25% of body weight bearing is applied; 50%, assisted gait in which a 50% of body weight bearing is applied.
