# Supplementary material for: Influence of the load exerted over a forearm crutch in spatiotemporal step parameters during assisted gait: pilot study
Source: Biomed Eng Online. 2018 Jul 18;17:98. doi: 10.1186/s12938-018-0527-z (PMC6052579; doi:10.1186/s12938-018-0527-z)
Supplement: Supplementary file 7 — Additional file 7. General comparisons between the loads applied to the crutch, for each study variable. [file 12938_2018_527_MOESM7_ESM.docx]

**Additional File 7 General comparisons between the loads applied to the crutch, for each study variable**

| **Parameter** | **NG-C** | | | **NG-25%** | | |
| --- | --- | --- | --- | --- | --- | --- |
|  | CI of the difference of means | P | Effect size | CI of the difference of means | P | Effect size |
| Il step length | -0.008;0.017 | Ns |  | 0.015;0.051 | 0.002 | 0.277 |
| Cl step length | 0.032;0.057 | <0.001 | 0.506 | 0.043;0.076 | <0.001 | 0.540 |
| Il step period | -0.134;-0.110 | <0.001 | 0.867 | -0.216;-0.169 | <0,001 | 0.868 |
| Cl step period | -0.110;-0.083 | <0.001 | 0.836 | -0.177;-0.113 | <0.001 | 0.662 |
| Velocity | 0.163;0.209 | <0.001 | 0.851 | 0.250;0.318 | <0.001 | 0.851 |
| Il step angle | 0.836;3.060 | 0,001 | 0.269 | 1.950;4.637 | <0.001 | 0.406 |
| Cl step angle | -3.275;-0.745 | 0.009 | 0.224 | -1.426;1.130 | Ns |  |
| Step width | -1.817;-0.013 | Ns |  | -1.640;0.108 | Ns |  |
|  | **NG-50%** | | | **C-25%** | | |
| Il step length | 0.026;0.064 | <0.001 | 0.359 | 0.021;0.041 | <0.001 | 0.485 |
| Cl step length | 0.061;0.101 | <0.001 | 0.578 | 0.001;0.024 | 0.002 | 0.287 |
| Il step period | -0.301;-0.240 | <0.001 | 0.866 | -0.095;-0.059 | <0.001 | 0.697 |
| Cl step period | -0.266;-0.206 | <0.001 | 0.853 | -0.086;-0.029 | <0.001 | 0.411 |
| Velocity | 0.329;0.392 | <0.001 | 0.868 | 0.089;0.128 | <0.001 | 0.762 |
| Il step angle | 1.913;4.405 | <0.001 | 0.408 | 0.260;1.789 | 0.037 | 0.191 |
| Cl step angle | -0.841;2.104 | Ns |  | 0.393;1.393 | 0.001 | 0.286 |
| Step width | -1.517;0.102 | Ns |  | -0.281;0.720 | Ns |  |
|  | **C-50%** | | | **25%-50%** | | |
| Il step length | 0.029;0.051 | <0.001 | 0.533 | 0.000;0.021 | Ns |  |
| Cl step length | 0.020;0.049 | <0.001 | 0.462 | 0.009;0.033 | <0.001 | 0.326 |
| Il step period | -0.170;-0.118 | <0.001 | 0.786 | -0.097;-0.059 | <0.001 | 0.668 |
| Cl step period | -0.162;-0.110 | <0.001 | 0.752 | -0.118;-0.061 | <0.001 | 0.519 |
| Velocity | 0.148;0.191 | <0.001 | 0.861 | 0.053;0.087 | <0.001 | 0.611 |
| Il step angle | 0.234;1.823 | 0.016 | 0.211 | -1.085;0.455 | Ns |  |
| Cl step angle | 0.968;2.371 | <0.001 | 0.409 | 0.086;1.473 | 0.028 | 0.078 |
| Step width | -0.243;0.799 | Ns |  | -0.108;0.225 | Ns |  |

NG, normal gait; C, assisted gait in which a comfortable load is applied; 25%, assisted gait in which a 25% of body weight bearing is applied; 50%, assisted gait in which a 50% of body weight bearing is applied; Il, ipsilateral; Cl, contralateral; CI, confidence interval; Ns, not significant.
