# Supplementary material for: Influence of the load exerted over a forearm crutch in spatiotemporal step parameters during assisted gait: pilot study
Source: Biomed Eng Online. 2018 Jul 18;17:98. doi: 10.1186/s12938-018-0527-z (PMC6052579; doi:10.1186/s12938-018-0527-z)
Supplement: Supplementary file 8 — Additional file 8. Representation of general comparisons between the loads applied to the crutch, for each study variable. [file 12938_2018_527_MOESM8_ESM.docx]

**Additional File 8 Representation of general comparisons between the loads applied to the crutch, for each study variable**


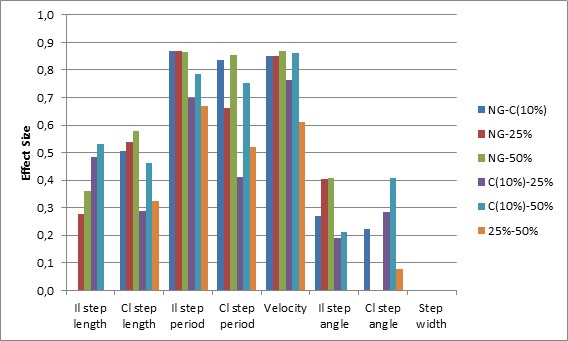


NG, normal gait; C, assisted gait in which a comfortable load is applied; 25%, assisted gait in which a 25% of body weight bearing is applied; 50%, assisted gait in which a 50% of body weight bearing is applied; Il, ipsilateral; Cl, contralateral.
