# Supplementary material for: Influence of the load exerted over a forearm crutch in spatiotemporal step parameters during assisted gait: pilot study
Source: Biomed Eng Online. 2018 Jul 18;17:98. doi: 10.1186/s12938-018-0527-z (PMC6052579; doi:10.1186/s12938-018-0527-z)
Supplement: Supplementary file 9 — Additional file 9. Step length analysis: difference of means between normal gait and unilateral assisted gait modalities (C, 25% and 50%). [file 12938_2018_527_MOESM9_ESM.docx]

**Additional File 9 Step length analysis: difference of means between normal gait and unilateral assisted gait modalities (C, 25% and 50%)**

|  | **Step length** | | | | | | | | |
| --- | --- | --- | --- | --- | --- | --- | --- | --- | --- |
| **Subject** | **Ipsilateral step length** | | | | | | | | |
|  | **NG-C** | | | **NG-25%** | | | **NG-50%** | | |
|  | CI of the difference of means (m) | P | Effect size | CI of the difference of means (m) | p | Effect size | CI of the difference of means (m) | p | Effect size |
| 1 | 0.044;0.132 | 0.001 | 0.700 | 0.078;0.178 | 0.005 | 0.886 | 0.130;0.198 | <0.001 | 0.901 |
| 2 | -0.024;0.054 | Ns |  | -0.006;0.060 | Ns |  | 0.088;0.138 | <0.001 | 0.905 |
| 3 | -0.080;-0.066 | <0.001 | 0.972 | -0.087;-0.063 | 0.005 | 0.887 | -0.109;-0.058 | <0.001 | 0.817 |
| 4 | -0.011;0.003 | Ns |  | 0.076;0.133 | 0.005 | 0.886 | 0.061;0.148 | <0.001 | 0.760 |
| 5 | 0.017;0.041 | <0.001 | 0.608 | 0.132;0.182 | <0.001 | 0.965 | 0.100;0.163 | <0.001 | 0.908 |
| 6 | -0.078;-0.039 | <0.001 | 0.767 | -0.081;-0.037 | <0.001 | 0.759 | -0.088;-0.034 | 0.001 | 0.763 |
| 7 | -0.108;-0.053 | 0.005 | 0.889 | -0.097;-0.055 | 0.004 | 0.907 | -0.111;-0.063 | 0.005 | 0.887 |
| 8 | -0.031;0.027 | Ns |  | -0.042;0.003 | Ns |  | -0.030;0.011 | Ns |  |
| 9 | -0.032;0.003 | Ns |  | -0.020;0.034 | Ns |  | -0.008;0.059 | Ns |  |
| 10 | 0.071;0.131 | <0.001 | 0.883 | 0.057;0.141 | <0.001 | 0.823 | 0.042;0.113 | 0.001 | 0.789 |
| 11 | 0.093;0.161 | <0.001 | 0.905 | 0.133;0.208 | 0.005 | 0.886 | 0.200;0.252 | <0.001 | 0.970 |
| **Subject** | **C-25%** | | | **C-50%** | | | **25%-50%** | | |
| 1 | -0.008;0.088 | Ns |  | 0.045;0.108 | <0.001 | 0.783 | 0.002;0.071 | 0.059 |  |
| 2 | -0.014;0.037 | Ns |  | 0.064;0.130 | <0.001 | 0.784 | 0.058;0.114 | <0.001 | 0.760 |
| 3 | -0.011;0.008 | Ns |  | -0.040;0.020 | Ns |  | -0.043;0.026 | Ns |  |
| 4 | 0.081;0.135 | 0.005 | 0.886 | 0.067;0.151 | 0.005 | 0.886 | -0.062;0.063 | Ns |  |
| 5 | 0.101;0.155 | <0.001 | 0.946 | 0.069;0.136 | <0.001 | 0.858 | -0.065;0.014 | Ns |  |
| 6 | -0.017;0.016 | Ns |  | -0.018;0.014 | Ns |  | -0.013;0.009 | Ns |  |
| 7 | -0.031;0.039 | Ns |  | -0.042;0.028 | Ns |  | -0.042;0.019 | Ns |  |
| 8 | -0.059;0.024 | Ns |  | -0.033;0.019 | Ns |  | -0.029;0.050 | Ns |  |
| 9 | -0.001;0.044 | Ns |  | 0.017;0.064 | 0.004 | 0.599 | -0.019;0.056 | Ns |  |
| 10 | -0.028;0.024 | Ns |  | -0.046;0.000 | 0.047 | 0.518 | -0.038;-0.004 | 0.022 | 0.346 |
| 11 | 0.011;0.075 | 0.022 | 0.725 | 0.067;0.131 | <0.001 | 0.864 | 0.020;0.091 | 0.009 | 0.822 |
| **Subject** | **Contralateral step length** | | | | | | | | |
|  | **NG-C** | | | **NG-25%** | | | **NG-50%** | | |
|  | CI of the difference of means (m) | P | Effect size | CI of the difference of means (m) | p | Effect size | CI of the difference of means (m) | p | Effect size |
| 1 | 0.032;0.122 | 0.004 | 0.703 | 0.092;0.190 | <0.001 | 0.871 | 0.136;0.200 | <0.001 | 0.899 |
| 2 | 0.039;0.081 | <0.001 | 0.838 | 0.061;0.111 | <0.001 | 0.870 | 0.107;0.155 | <0.001 | 0.943 |
| 3 | -0.065;-0.026 | 0.005 | 0.887 | -0.092;-0.063 | 0.005 | 0.887 | -0.083;-0.042 | 0.005 | 0.887 |
| 4 | -0.003;0.015 | Ns |  | 0.075;0.095 | <0.001 | 0.945 | 0.070;0.129 | <0.001 | 0.835 |
| 5 | -0.029;0.023 | Ns |  | 0.008;0.051 | 0.012 | 0.470 | 0.030;0.100 | 0.002 | 0.728 |
| 6 | -0.013;0.017 | Ns |  | -0.013;0.026 | Ns |  | -0.027;0.014 | Ns |  |
| 7 | -0.053;0.103 | Ns |  | -0.056;-0.043 | 0.004 | 0.907 | -0.091;-0.067 | 0.005 | 0.887 |
| 8 | -0.006;0.050 | Ns |  | 0.004;0.046 | 0.023 | 0.512 | -0.027;0.120 | Ns |  |
| 9 | 0.028;0.147 | 0.005 | 0.886 | 0.022;0.084 | 0.009 | 0.822 | 0.015;0.210 | 0.005 | 0.886 |
| 10 | 0.109;0.173 | 0.005 | 0.887 | 0.147;0.213 | <0.001 | 0.932 | 0.191;0.230 | <0.001 | 0.953 |
| 11 | 0.134;0.185 | <0.001 | 0.935 | 0.143;0.223 | <0.001 | 0.952 | 0.200;0.285 | <0.001 | 0.956 |
| **Subject** | **C-25%** | | | **C-50%** | | | **25%-50%** | | |
| 1 | 0.048;0.080 | <0.001 | 0.835 | 0.074;0.128 | <0.001 | 0.878 | 0.009;0.065 | 0.015 | 0.549 |
| 2 | 0.000;0.052 | 0.047 | 0.475 | 0.052;0.091 | <0.001 | 0.840 | 0.015;0.075 | 0.008 | 0.638 |
| 3 | -0.037;-0.026 | 0.004 | 0.907 | -0.037;0.003 | Ns |  | -0.002;0.031 | Ns |  |
| 4 | 0.066;0.093 | 0.005 | 0.886 | 0.062;0.126 | 0.005 | 0.886 | -0.021;0.050 | Ns |  |
| 5 | -0.008;0.074 | Ns |  | 0.031;0.106 | 0.007 | 0.854 | -0.010;0.081 | Ns |  |
| 6 | -0.007;0.016 | Ns |  | -0.024;0.008 | Ns |  | -0.025; 0.000 | Ns |  |
| 7 | -0.151;0.003 | Ns |  | -0.179;-0.029 | 0.005 | 0.887 | -0.045;-0.014 | 0.007 | 0.855 |
| 8 | -0.026;0.033 | Ns |  | -0.046;0.095 | Ns |  | -0.046;0.089 | Ns |  |
| 9 | -0.089;0.019 | Ns |  | -0.035;0.084 | Ns |  | -0.035;0.155 | Ns |  |
| 10 | 0.023;0.055 | 0.007 | 0.854 | 0.044;0.094 | 0.005 | 0.889 | 0.009;0.052 | 0.011 | 0.485 |
| 11 | -0.007;0.054 | Ns |  | 0.043;0.121 | 0.001 | 0.739 | 0.019;0.099 | 0.009 | 0.625 |

NG, normal gait, C, assisted gait in which a comfortable load is applied; 25%, assisted gait in which a 25% of body weight bearing is applied; 50%, assisted gait in which a 50% of body weight bearing is applied; CI, confidence interval; Ns, not significant.
