# Supplementary material for: Influence of the load exerted over a forearm crutch in spatiotemporal step parameters during assisted gait: pilot study
Source: Biomed Eng Online. 2018 Jul 18;17:98. doi: 10.1186/s12938-018-0527-z (PMC6052579; doi:10.1186/s12938-018-0527-z)
Supplement: Supplementary file 10 — Additional file 10. Step period analysis: difference of means between gait without crutches and unilateral assisted gait modalities (C, 25% and 50%). [file 12938_2018_527_MOESM10_ESM.docx]

**Additional File 10 Step period analysis: difference of means between gait without crutches and unilateral assisted gait modalities (C, 25% and 50%)**

|  | **Step period** | | | | | | | | |
| --- | --- | --- | --- | --- | --- | --- | --- | --- | --- |
| **Subject** | **Ipsilateral step period** | | | | | | | | |
|  | **NG-C** | | | **NG-25%** | | | **NG-50%** | | |
|  | CI of the difference of means (m) | P | Effect size | CI of the difference of means (m) | p | Effect size | CI of the difference of means (m) | p | Effect size |
| 1 | -0.169;-0.118 | <0.001 | 0.956 | -0.435;-0.370 | <0.001 | 0.981 | -0.539;-0.420 | <0.001 | 0.969 |
| 2 | -0.157;-0.114 | <0.001 | 0.939 | -0.271;-0.210 | <0.001 | 0.966 | -0.368;-0.282 | <0.001 | 0.954 |
| 3 | -0.068;-0.055 | 0.005 | 0.887 | -0.057;-0.044 | 0.005 | 0.887 | -0.178;-0.128 | <0.001 | 0.956 |
| 4 | -0.073;-0.024 | 0.007 | 0.855 | -0.232;-0.183 | 0.005 | 0.886 | -0.374;-0.196 | 0.007 | 0.854 |
| 5 | -0.078;-0.049 | <0.001 | 0.853 | -0.089;-0.033 | 0.001 | 0.788 | -0.084;-0.023 | 0.003 | 0.642 |
| 6 | -0.119;-0.093 | 0.005 | 0.889 | -0.140;-0.113 | <0.001 | 0.950 | -0.191;-0.171 | 0.005 | 0.889 |
| 7 | -0.193;-0.135 | 0.005 | 0.889 | -0.206;-0.177 | 0.004 | 0.907 | -0.234;-0.174 | 0.005 | 0.887 |
| 8 | -0.135;-0.080 | <0.001 | 0.834 | -0.172;-0.117 | <0.001 | 0.890 | -0.223;-0.136 | <0.001 | 0.921 |
| 9 | -0.177;-0.111 | <0.001 | 0.913 | -0.247;-0.164 | <0.001 | 0.939 | -0.269;-0.147 | <0.001 | 0.898 |
| 10 | -0.063;-0.036 | <0.001 | 0.888 | -0.097;-0.044 | <0.001 | 0.856 | -0.121;-0.076 | <0.001 | 0.927 |
| 11 | -0.278;-0.176 | <0.001 | 0.914 | -0.525;-0.419 | <0.001 | 0.974 | -0.754;-0.580 | <0.001 | 0.974 |
| **Subject** | **C-25%** | | | **C-50%** | | | **25%-50%** | | |
| 1 | -0.307;-0.211 | <0.001 | 0.953 | -0.396;-0.276 | <0.001 | 0.938 | -0.141;-0.013 | 0.024 | 0.479 |
| 2 | -0.133;-0.077 | <0.001 | 0.854 | -0.244;-0.136 | <0.001 | 0.880 | -0.149;-0.021 | 0.015 | 0.610 |
| 3 | 0.008;0.013 | 0.004 | 0.907 | -0.114;-0.069 | 0.005 | 0.898 | -0.127;-0.077 | 0.005 | 0.898 |
| 4 | -0.192;-0.125 | 0.005 | 0.886 | -0.303;-0.169 | 0.007 | 0.854 | -0.170;0.015 | Ns |  |
| 5 | -0.026;0.030 | Ns |  | -0.026;0.045 | Ns |  | -0.038;0.053 | Ns |  |
| 6 | -0.042;0.001 | Ns |  | -0.087;-0.063 | 0.004 | 0.907 | -0.076;-0.033 | 0.005 | 0.889 |
| 7 | -0.063;0.008 | Ns |  | -0.82;0.001 | Ns |  | -0.049;0.024 | Ns |  |
| 8 | -0.078;0.004 | Ns |  | -0.114;-0.030 | 0.004 | 0.687 | -0.063;-0.005 | 0.026 | 0.401 |
| 9 | -0.087;-0.036 | <0.001 | 0.782 | -0.109;-0.019 | 0.010 | 0.604 | -0.049;0.044 | Ns |  |
| 10 | -0.038;-0.003 | 0.025 | 0.499 | -0.063;-0.034 | <0.001 | 0.831 | -0.037;-0.019 | <0.001 | 0.509 |
| 11 | -0.293;-0.197 | <0.001 | 0.873 | -0.539;-0.341 | <0.001 | 0.929 | -0.271;-0.120 | <0.001 | 0.736 |
| **Subject** | **Contralateral step period** | | | | | | | | |
|  | **NG-C** | | | **NG-25%** | | | **NG-50%** | | |
|  | CI of the difference of means (m) | P | Effect size | CI of the difference of means (m) | p | Effect size | CI of the difference of means (m) | p | Effect size |
| 1 | -0.188;-0.131 | <0.001 | 0.935 | -0.425;-0.335 | <0.001 | 0.973 | -0.497;-0.398 | <0.001 | 0.968 |
| 2 | -0.157;-0.114 | <0.001 | 0.939 | -0.271;-0.210 | <0.001 | 0.966 | -0.368;-0.282 | <0.001 | 0.954 |
| 3 | -0.053;-0.020 | 0.005 | 0.887 | -0.069;-0.038 | 0.005 | 0.887 | -0.153;-0.112 | 0.005 | 0.887 |
| 4 | -0.060;-0.017 | 0.012 | 0.790 | -0.279;-0.200 | 0.005 | 0.886 | -0.334;-0.259 | 0.005 | 0.886 |
| 5 | -0.118;-0.068 | <0.001 | 0.897 | -0.226;-0.186 | <0.001 | 0.960 | -0.158;-0.092 | <0.001 | 0.875 |
| 6 | -0.067;-0.035 | <0.001 | 0.865 | -0.075;-0.051 | <0.001 | 0.849 | -0.116;-0.093 | 0.005 | 0.889 |
| 7 | -0.141;0.019 | Ns |  | -0.171;-0.158 | 0.004 | 0.907 | -0.217;-0.174 | 0.005 | 0.887 |
| 8 | -0.128;-0.062 | <0.001 | 0.820 | -0.157;0.041 | Ns |  | -0.186;-0.100 | <0.001 | 0.893 |
| 9 | -0.111;-0.054 | 0.005 | 0.887 | -0.138;0.100 | Ns |  | -0.165;-0.084 | <0.001 | 0.866 |
| 10 | -0.026;0.006 | Ns |  | -0.015;0.037 | Ns |  | 0.017;0.057 | 0.002 | 0.620 |
| 11 | -0.227;-0.124 | <0.001 | 0.885 | -0.548;-0.355 | <0.001 | 0.926 | -0.684;-0.546 | <0.001 | 0.976 |
| **Subject** | **C-25%** | | | **C-50%** | | | **25%-50%** | | |
| 1 | -0.276;-0.166 | <0.001 | 0.909 | -0.345;-0.232 | <0.001 | 0.917 | -0.136;0.001 | Ns |  |
| 2 | -0.133;-0.077 | <0.001 | 0.854 | -0.244;-0.136 | <0.001 | 0.880 | -0.149;-0.021 | 0.015 | 0.610 |
| 3 | -0.021;-0.014 | 0.004 | 0.907 | -0.123;-0.070 | 0.005 | 0.898 | -0.106;-0.051 | 0.005 | 0.898 |
| 4 | -0.233;-0.168 | 0.005 | 0.886 | -0.307;-0.209 | 0.005 | 0.886 | -0.115;-0.001 | 0.048 | 0.453 |
| 5 | -0.152;-0.074 | <0.001 | 0.863 | -0.069;0.004 | Ns |  | 0.036;0.126 | 0.003 | 0.693 |
| 6 | -0.026;0.002 | Ns |  | -0.071;-0.036 | 0.004 | 0.907 | -0.062;-0.022 | 0.005 | 0.889 |
| 7 | -0.188;-0.019 | 0.007 | 0.856 | -0.226;-0.043 | 0.005 | 0.887 | -0.051;-0.011 | 0.007 | 0.568 |
| 8 | -0.062;0.136 | Ns |  | -0.081;-0.016 | 0.009 | 0.582 | -0.179;0.009 | Ns |  |
| 9 | -0.058;0.185 | Ns |  | -0.067;-0.018 | 0.004 | 0.686 | -0.229;0.018 | Ns |  |
| 10 | 0.005;0.038 | 0.013 | 0.790 | 0.025;0.069 | 0.007 | 0.856 | 0.002;0.050 | 0.036 | 0.440 |
| 11 | -0.349;-0.204 | <0.001 | 0.813 | -0.529;-0.350 | <0.001 | 0.944 | -0.302;-0.025 | 0.026 | 0.593 |

NG, normal gait, C, assisted gait in which a comfortable load is applied; 25%, assisted gait in which a 25% of body weight bearing is applied; 50%, assisted gait in which a 50% of body weight bearing is applied; CI, confidence interval; Ns, not significant.
