# Supplementary material for: Influence of the load exerted over a forearm crutch in spatiotemporal step parameters during assisted gait: pilot study
Source: Biomed Eng Online. 2018 Jul 18;17:98. doi: 10.1186/s12938-018-0527-z (PMC6052579; doi:10.1186/s12938-018-0527-z)
Supplement: Supplementary file 11 — Additional file 11. Gait velocity analysis: difference of means between gait without crutches and unilateral assisted gait modalities (C, 25% and 50%). [file 12938_2018_527_MOESM11_ESM.docx]

**Additional File 11 Gait velocity analysis: difference of means between gait without crutches and unilateral assisted gait modalities (C, 25% and 50%)**

| **Subject** | **Velocity** | | | | | | | | |
| --- | --- | --- | --- | --- | --- | --- | --- | --- | --- |
|  | **NG-C** | | | **NG-25%** | | | **NG-50%** | | |
|  | CI of the difference of means (m) | P | Effect size | CI of the difference of means (m) | p | Effect size | CI of the difference of means (m) | p | Effect size |
| 1 | 0.251;0.384 | <0.001 | 0.933 | 0.487;0.611 | <0.001 | 0.982 | 0.566;0.669 | <0.001 | 0.985 |
| 2 | 0.166;0.252 | <0.001 | 0.918 | 0.281;0.367 | <0.001 | 0.969 | 0.448;0.515 | <0.001 | 0.990 |
| 3 | -0.029;0.007 | Ns |  | -0.055;-0.009 | 0.012 | 0.790 | 0.094;0.133 | 0.005 | 0.887 |
| 4 | 0.034;0.097 | 0.007 | 0.855 | 0.328;0.407 | 0.005 | 0.886 | 0.369;0.469 | 0.005 | 0.886 |
| 5 | 0.166;0.203 | <0.001 | 0.947 | 0.378;0.413 | <0.001 | 0.991 | 0.320;0.363 | <0.001 | 0.986 |
| 6 | 0.033;0.129 | 0.009 | 0.824 | 0.083;0.166 | 0.005 | 0.889 | 0.089;0.194 | 0.005 | 0.889 |
| 7 | 0.133;0.192 | 0.005 | 0.889 | 0.200;0.220 | 0.004 | 0.907 | 0.182;0.237 | 0.005 | 0.887 |
| 8 | 0.097;0.229 | 0.005 | 0.886 | 0.117;0.243 | 0.005 | 0.889 | 0.186;0.287 | 0.005 | 0.887 |
| 9 | 0.107;0.229 | 0.007 | 0.855 | 0.190;0.318 | 0.005 | 0.886 | 0.206;0.350 | 0.005 | 0.886 |
| 10 | 0.236;0.285 | 0.005 | 0.887 | 0.255;0.325 | 0.005 | 0.886 | 0.276;0.323 | <0.001 | 0.986 |
| 11 | 0.373;0.508 | 0.005 | 0.886 | 0.550;0.694 | 0.005 | 0.886 | 0.676;0.793 | <0.001 | 0.990 |
| **Subject** | **C-25%** | | | **C-50%** | | | **25%-50%** | | |
| 1 | 0.185;0.278 | <0.001 | 0.940 | 0.257;0.343 | <0.001 | 0.963 | 0.042;0.094 | <0.001 | 0.752 |
| 2 | 0.079;0.151 | <0.001 | 0.751 | 0.246;0.298 | <0.001 | 0.950 | 0.118;0.197 | <0.001 | 0.886 |
| 3 | -0.033;-0.009 | 0.011 | 0.808 | 0.109;0.141 | 0.005 | 0.898 | 0.126;0.166 | 0.005 | 0.898 |
| 4 | 0.278;0.325 | 0.005 | 0.886 | 0.317;0.389 | 0.005 | 0.886 | 0.005;0.098 | 0.033 | 0.523 |
| 5 | 0.189;0.233 | <0.001 | 0.972 | 0.129;0.185 | <0.001 | 0.947 | -0.070;-0.038 | <0.001 | 0.783 |
| 6 | 0.026;0.061 | 0.005 | 0.889 | 0.054;0.068 | 0.004 | 0.907 | -0.003;0.037 | Ns |  |
| 7 | 0.018;0.078 | 0.012 | 0.792 | 0.008;0.086 | 0.028 | 0.693 | -0.026;0.024 | Ns |  |
| 8 | -0.078;0.112 | Ns |  | 0.026;0.122 | 0.007 | 0.620 | -0.002;0.115 | Ns |  |
| 9 | 0.046;0.126 | 0.009 | 0.822 | 0.073;0.148 | 0.005 | 0.886 | -0.037;0.084 | Ns |  |
| 10 | 0.000;0.058 | 0.059 |  | 0.019;0.059 | 0.007 | 0.856 | -0.019;0.038 | Ns |  |
| 11 | 0.158;0.206 | 0.005 | 0.886 | 0.262;0.327 | 0.005 | 0.886 | 0.074;0.151 | 0.005 | 0.886 |

NG, normal gait, C, assisted gait in which a comfortable load is applied; 25%, assisted gait in which a 25% of body weight bearing is applied; 50%, assisted gait in which a 50% of body weight bearing is applied; CI, confidence interval; Ns, not significant.
