# Supplementary material for: Influence of the load exerted over a forearm crutch in spatiotemporal step parameters during assisted gait: pilot study
Source: Biomed Eng Online. 2018 Jul 18;17:98. doi: 10.1186/s12938-018-0527-z (PMC6052579; doi:10.1186/s12938-018-0527-z)
Supplement: Supplementary file 12 — Additional file 12. Step angle analysis: difference of means between gait without crutches and unilateral assisted gait modalities (C, 25% and 50%). [file 12938_2018_527_MOESM12_ESM.docx]

**Additional File 12 Step angle analysis: difference of means between gait without crutches and unilateral assisted gait modalities (C, 25% and 50%)**

|  | **Step angle** | | | | | | | | |
| --- | --- | --- | --- | --- | --- | --- | --- | --- | --- |
| **Subject** | **Ipsilateral step angle** | | | | | | | | |
|  | **NG-C** | | | **NG-25%** | | | **NG-50%** | | |
|  | CI of the difference of means (m) | P | Effect size | CI of the difference of means (m) | p | Effect size | CI of the difference of means (m) | p | Effect size |
| 1 | 1.655;8.893 | 0.009 | 0.525 | -0.843;10.616 | Ns |  | 1.461;9.291 | Ns |  |
| 2 | 8.390;16.587 | <0.001 | 0.883 | 7.314;12.908 | <0.001 | 0.848 | 6.477;12.343 | <0.001 | 0.812 |
| 3 | -3.396;2.055 | Ns |  | -2.621;1.906 | Ns |  | -2.010;3.291 | Ns |  |
| 4 | -2.220;0.099 | Ns |  | -2.095;1.840 | Ns |  | -3.712;1.297 | Ns |  |
| 5 | -0.975;6.364 | Ns |  | 3.865;11.946 | 0.002 | 0.716 | 0.765;8.926 | 0.025 | 0.492 |
| 6 | -13.006;-3.597 | 0.021 | 0.727 | -10.192;0.242 | Ns |  | -8.306;1.069 | Ns |  |
| 7 | -0.824;4.094 | Ns |  | -0.420;1.481 | Ns |  | -0.362;3.322 | Ns |  |
| 8 | -1.919;7.121 | Ns |  | 1.898;11.081 | 0.011 | 0.628 | 2.666;10.756 | 0.005 | 0.681 |
| 9 | -0.162;4.267 | Ns |  | -1.505;5.624 | Ns |  | -1.473;6.330 | Ns |  |
| 10 | 3.079;7.682 | 0.001 | 0.711 | 3.718;10.396 | 0.001 | 0.764 | 3.304;7.640 | <0.001 | 0.669 |
| 11 | 9.342;15.614 | <0.001 | 0.920 | 8.897;15.325 | <0.001 | 0.891 | 8.019;14.399 | <0.001 | 0.867 |
| **Subject** | **C-25%** | | | **C-50%** | | | **25%-50%** | | |
| 1 | -4.563;3.789 | Ns |  | -3.448;3.652 | Ns |  | -2.095;3.073 | Ns |  |
| 2 | -4.757;0.003 | Ns |  | -6.156;0.000 | 0.050 | 0.450 | -2.548;1.146 | Ns |  |
| 3 | -0.764;1.390 | Ns |  | -1.327;3.949 | Ns |  | -1.840;3.835 | Ns |  |
| 4 | -0.872;2.738 | Ns |  | -2.415;2.121 | Ns |  | -3.983;1.824 | Ns |  |
| 5 | 1.437;8.985 | 0.012 | 0.494 | -1.534;5.836 | Ns |  | -6.741;0.622 | Ns |  |
| 6 | -0.036;6.688 | Ns |  | 2.669;6.696 | 0.006 | 0.874 | -2.485;5.198 | Ns |  |
| 7 | -3.784;1.575 | Ns |  | -3.086;2.777 | Ns |  | -1.580;3.480 | Ns |  |
| 8 | -0.458;8.235 | Ns |  | 1.070;7.149 | 0.014 | 0.435 | -3.378;3.820 | Ns |  |
| 9 | -1.754;1.769 | Ns |  | -2.179;2.931 | Ns |  | -2.525;3.262 | Ns |  |
| 10 | -0.512;3.864 | Ns |  | -1.847;2.031 | Ns |  | -3.925;0.755 | Ns |  |
| 11 | -2.924;2.190 | Ns |  | -5.445;2.908 | Ns |  | -5.100;3.296 | Ns |  |
| **Subject** | **Contralateral step angle** | | | | | | | | |
|  | **NG-C** | | | **NG-25%** | | | **NG-50%** | | |
|  | CI of the difference of means (m) | P | Effect size | CI of the difference of means (m) | p | Effect size | CI of the difference of means (m) | p | Effect size |
| 1 | -2.453;4.242 | Ns |  | -2.692;3.782 | Ns |  | -1.431;4.416 | Ns |  |
| 2 | 2.633;10.438 | 0.004 | 0.733 | 6.258;15.083 | <0.001 | 0.841 | 10.835;17.942 | <0.001 | 0.924 |
| 3 | -3.824;0.514 | Ns |  | -0.380;0.569 | Ns |  | -7.846;1.410 | Ns |  |
| 4 | -9.700;-7.675 | 0.005 | 0.887 | -10.480;-8.675 | <0.001 | 0.975 | -10.229;-7.862 | <0.001 | 0.923 |
| 5 | 1.907;6.382 | 0.002 | 0.655 | 1.258;6.824 | 0.009 | 0.628 | 3.654;9.350 | 0.001 | 0.787 |
| 6 | -12.553;-7.000 | 0.005 | 0.887 | -13.167;-7.194 | 0.005 | 0.887 | -12.786;-6.690 | 0.005 | 0.886 |
| 7 | 0.343;3.843 | 0.012 | 0.792 | 3.689;7.208 | <0.001 | 0.843 | -1.849;7.312 | Ns |  |
| 8 | -1.020;3.562 | Ns |  | 0.095;4.581 | 0.047 | 0.629 | 0.875;5.105 | 0.022 | 0.725 |
| 9 | -2.267;3.761 | Ns |  | -0.436;4.962 | Ns |  | -1.080;6.511 | Ns |  |
| 10 | -3.315;2.399 | Ns |  | -3.813;1.871 | Ns |  | -3.811;1.744 | Ns |  |
| 11 | 3.676;10.009 | 0.001 | 0.794 | 3.325;9.811 | 0.001 | 0.711 | 9.136;13.396; | <0.001 | 0.904 |
| **Subject** | **C-25%** | | | **C-50%** | | | **25%-50%** | | |
| 1 | -2.226;1.527 | Ns |  | -1.239;2.435 | Ns |  | -1.253;3.148 | Ns |  |
| 2 | 1.545;6.725 | 0.006 | 0.592 | 6.071;9.635 | <0.001 | 0.868 | 1.331;6.105 | 0.006 | 0.558 |
| 3 | -0.452;0.952 | Ns |  | -5.134;2.008 | Ns |  | -5.642;2.017 | Ns |  |
| 4 | -2.314;0.535 | Ns |  | -2.032;1.316 | Ns |  | -0.988;2.051 | Ns |  |
| 5 | -1.123;0.916 | Ns |  | 0.944;3.771 | 0.004 | 0.592 | 0.710;4.212 | 0.011 | 0.572 |
| 6 | -1.726;0.918 | Ns |  | -2.473;2.550 | Ns |  | -1.897;2.781 | Ns |  |
| 7 | 0.507;6.204 | 0.021 | 0.727 | -4.201;5.478 | Ns |  | -8.739;3.304 | Ns |  |
| 8 | -0.848;2.982 | Ns |  | -0.164;3.603 | Ns |  | -0.834;2.139 | Ns |  |
| 9 | 0.190;2.842 | 0.029 | 0.494 | 0.213;3.725 | 0.032 | 0.480 | -1.491;2.396 | Ns |  |
| 10 | -1.359;0.334 | Ns |  | -1.972;0.820 | Ns |  | -0.966;0.840 | Ns |  |
| 11 | -2.838;2.288 | Ns |  | 2.124;6.723 | 0.002 | 0.650 | 2.584;6.812 | 0.001 | 0.589 |

NG, normal gait, C, assisted gait in which a comfortable load is applied; 25%, assisted gait in which a 25% of body weight bearing is applied; 50%, assisted gait in which a 50% of body weight bearing is applied; CI, confidence interval; Ns, not significant.
