# Supplementary material for: Influence of the load exerted over a forearm crutch in spatiotemporal step parameters during assisted gait: pilot study
Source: Biomed Eng Online. 2018 Jul 18;17:98. doi: 10.1186/s12938-018-0527-z (PMC6052579; doi:10.1186/s12938-018-0527-z)
Supplement: Supplementary file 13 — Additional file 13. Step width analysis: difference of means between gait without crutches and unilateral assisted gait modalities (C, 25% and 50%). [file 12938_2018_527_MOESM13_ESM.docx]

**Additional File 13 Step width analysis: difference of means between gait without crutches and unilateral assisted gait modalities (C, 25% and 50%)**

| **Subject** | **Step width** | | | | | | | | |
| --- | --- | --- | --- | --- | --- | --- | --- | --- | --- |
|  | **NG-C** | | | **NG-25%** | | | **NG-50%** | | |
|  | CI of the difference of means (m) | P | Effect size | CI of the difference of means (m) | p | Effect size | CI of the difference of means (m) | p | Effect size |
| 1 | -0.037;0.020 | Ns |  | -0.033;0.028 | Ns |  | -0.022;0.049 | Ns |  |
| 2 | -0.031;0.001 | Ns |  | -0.098;0.013 | Ns |  | -0.044;-0.015 | 0.001 | 0.727 |
| 3 | -0.004;0135 | Ns |  | -0.038;0.103 | Ns |  | -0.020;0.120 | Ns |  |
| 4 | -0.018;0.010 | Ns |  | -0.045;-0.017 | 0.009 | 0.822 | -0.066;-0.030 | 0.007 | 0.854 |
| 5 | 0.016;0.054 | 0.009 | 0.823 | 0.011;0.060 | 0.009 | 0.597 | -0.005;0.049 | Ns |  |
| 6 | -0.009;0.014 | Ns |  | -0.013;0.011 | Ns |  | -0.017;0.017 | Ns |  |
| 7 | -0.014;0.030 | Ns |  | 0.038;0.046 | 0.004 | 0.907 | -0.002;0.028 | Ns |  |
| 8 | -0.012;0.026 | Ns |  | -0.007;0.026 | Ns |  | 0.003;0.022 | 0.017 | 0.377 |
| 9 | -0.012;0.020 | Ns |  | -0.007;0.016 | Ns |  | -0.029;-0.001 | 0.074 |  |
| 10 | -0.013;0.031 | Ns |  | -0.012;0.024 | Ns |  | -0.005;0.036 | Ns |  |
| 11 | -0.030;0.020 | Ns |  | -0.008;0.035 | Ns |  | -0.031;0.016 | Ns |  |
| **Subject** | **C-25%** | | | **C-50%** | | | **25%-50%** | | |
| 1 | -0.019;0.032 | Ns |  | -0.007;0.051 | Ns |  | -0.002;0.033 | Ns |  |
| 2 | -0.089;0.035 | Ns |  | -0.031;0.002 | Ns |  | -0.049;0.074 | Ns |  |
| 3 | -0.037;-0.028 | 0.004 | 0.907 | -0.022;-0.008 | 0.005 | 0.898 | 0.010;0.025 | 0.006 | 0.866 |
| 4 | -0.035;-0.020 | 0.005 | 0.886 | -0.056;-0.033 | 0.005 | 0.886 | -0.033;0.000 | 0.059 |  |
| 5 | -0.018;0.020 | Ns |  | -0.029;0.004 | Ns |  | -0.025;-0.003 | 0.021 | 0.398 |
| 6 | -0.012;0.005 | Ns |  | -0.015;0.011 | Ns |  | -0.008;0.012 | Ns |  |
| 7 | 0.010;0.058 | 0.012 | 0.792 | -0.019;0.029 | Ns |  | -0.042;-0.017 | 0.007 | 0.855 |
| 8 | -0.011;0.017 | Ns |  | -0.009;0.021 | Ns |  | -0.007;0.013 | Ns |  |
| 9 | -0.012;0.013 | Ns |  | -0.032;-0.006 | 0.013 | 0.790 | -0.034;-0.005 | 0.015 | 0.563 |
| 10 | -0.022;0.016 | Ns |  | -0.012;0.024 | Ns |  | -0.003;0.022 | Ns |  |
| 11 | 0.005;0.032 | 0.014 | 0.514 | -0.021;0.015 | Ns |  | -0.037;-0.005 | 0.014 | 0.663 |

NG, normal gait, C, assisted gait in which a comfortable load is applied; 25%, assisted gait in which a 25% of body weight bearing is applied; 50%, assisted gait in which a 50% of body weight bearing is applied; CI, confidence interval; Ns, not significant.
