# Supplementary material for: Influence of the load exerted over a forearm crutch in spatiotemporal step parameters during assisted gait: pilot study
Source: Biomed Eng Online. 2018 Jul 18;17:98. doi: 10.1186/s12938-018-0527-z (PMC6052579; doi:10.1186/s12938-018-0527-z)
Supplement: Supplementary file 14 — Additional file 14. Representation of the difference between gait without crutches and unilateral assisted gait modalities (C, 25% and 50%) for each subject. [file 12938_2018_527_MOESM14_ESM.docx]

**Additional File 14 Representation of the difference between gait without crutches and unilateral assisted gait modalities (C, 25% and 50%) for each subject**

**
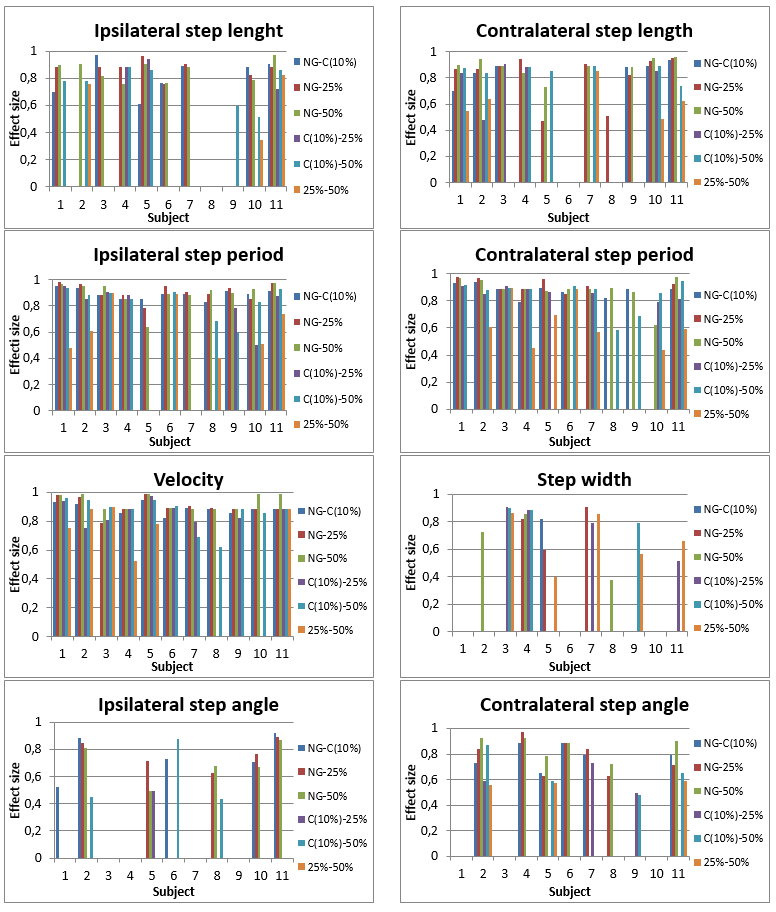
**

NG, normal gait; C, assisted gait in which a comfortable load is applied; 25%, assisted gait in which a 25% of body weight bearing is applied; 50%, assisted gait in which a 50% of body weight bearing is applied.
